# Dynamic landslide susceptibility analysis that combines rainfall period, accumulated rainfall, and geospatial information

**DOI:** 10.1038/s41598-022-21795-z

**Published:** 2022-11-01

**Authors:** Jae-Joon Lee, Moon-Soo Song, Hong-Sik Yun, Sang-Guk Yum

**Affiliations:** 1 Interdisciplinary Program in Crisis, Disaster and Risk Management, Sungkyunkwan University, Suwon, Gyeonggi-do Republic of Korea; 2 Department of Civil Engineering, Gangneung-Wonju National University, Gangneung, Gangwon-do Republic of Korea

**Keywords:** Natural hazards, Solid Earth sciences, Engineering

## Abstract

Worldwide, catastrophic landslides are occurring as a result of abnormal climatic conditions. Since a landslide is caused by a combination of the triggers of rainfall and the vulnerability of spatial information, a study that can suggest a method to analyze the complex relationship between the two factors is required. In this study, the relationship between complex factors (rainfall period, accumulated rainfall, and spatial information characteristics) was designed as a system dynamics model as variables to check the possibility of occurrence of vulnerable areas according to the rainfall characteristics that change in real-time. In contrast to the current way of predicting the collapse time by analysing rainfall data, the developed model can set the precipitation period during rainfall. By setting the induced rainfall period, the researcher can then assess the susceptibility of the landslide-vulnerable area. Further, because the geospatial information features and rainfall data for the 672 h before the landslide's occurrence were combined, the results of the susceptibility analysis could be determined for each topographical characteristic according to the rainfall period and cumulative rainfall change. Third, by adjusting the General cumulative rainfall period (D_G_) and Inter-event time definition (IETD), the preceding rainfall period can be adjusted, and desired results can be obtained. An analysis method that can solve complex relationships can contribute to the prediction of landslide warning times and expected occurrence locations.

## Introduction

Increased rainfall brought on by climate change triggers landslides at vulnerable locations, thus causing public damage. Therefore, a prominent global academic effort is underway with regard to disaster preparedness. The majority of research on landslide-occurrence prediction and susceptibility is divided into two categories: rainfall analysis and spatial information-based research. Rainfall analysis studies identify the rainfall thresholds based on regression analysis of rainfall intensity and period data. Spatial information-based research is based on utilizing the frequency ratio of susceptible factors, regression, and machine learning analysis.

The sensitivity function has been studied using critical rainfall^1^, and a relationship between the rainfall intensity and total cumulative rainfall has been suggested^2,3^ to predict landslide occurrence probability. Rainfall analysis can be used for alerting, monitoring, emergency-response planning, and other purposes^4,5^. Therefore, based on the literature review and researchers’ understanding, the rainfall thresholds were determined and examined while considering different rainfall conditions^1–4,6,7^.

Geographic information system (GIS) is mainly used for landslide analysis to support decision-making owing to its visual advantages. GIS-based landslide-related studies from 1999 to 2018 are the most widely used in contemporary research and applications^6^. Landslide susceptibility has been analysed using GIS to predict landslide occurrence based on spatial information such as geomorphology, geology, hydrology characteristics, and rainfall^7–13^. The relationship of geospatial information factors which affect the landslide has been evaluated using regression analysis and GIS technology^7,11,14–17^. These analyses primarily use a frequency-ratio method based on pixel units, which was achieved using artificial intelligence-based methods^8,18–20^ and MATLAB^21^. Research on the integration of machine learning and GIS technology is underway to increase forecast accuracy.

Convolutional neural network, deep neural network, long short-term memory, and recurrent neural network were used to forecast landslides using deep learning algorithms by analysing the frequency ratios of pixels^22^. Furthermore, predictions with 90% accuracy were produced by integrating K-nearest neighbour, multi-layer perceptron, random forest, and support vector machine^23^. Various analysis techniques are being tested using different conditions for points, circles, and polygons^24^. However, accuracy may differ based on the choice of non-landslide site. The key argument is that each location requires a different appropriate model and that using a single model across all regions is not feasible.

Rainfall was considered in conjunction with spatial information in previous studies^25^ because it is difficult to anticipate the time and location of occurrence solely using spatial information-based analysis. Landslide susceptibility caused by rainfall differs depending on weathered soil type^26^ and geomorphological characteristics^27^ of shallow landslides in Italy^28^. The dynamic susceptibility map for extreme rainfall changed by performing a logistic regression analysis based on rainfall and GIS in the Deokjeok-ri and Chuncheon regions in the Republic of Korea^7^. The National Disaster Management Research Institute in the Republic of Korea conducted a risk map study of rainfall by applying a three-dimensional limit equilibrium analysis model of cancer species and a soil depth prediction model to determine spatial characteristic information^29–31^. It is difficult for the aforementioned studies, which combine geographical and meteorological data, to adapt to real-time changes utilizing fixed variables. In this study, we performed probability-based modelling to understand the relationship between rainfall duration, amount of cumulative rainfall, and spatial information according to changes in rainfall conditions. The proposed method considers region-based rainfall characteristics to reflect dynamic rainfall factors. A linkage model between rainfall and spatial information and a probability model considering changes in the rainfall conditions of the geospatial information (GSI) were developed. As a consequence, it was feasible to detect the spatial information's detailed geospatial information (DGSI) susceptibility by modelling. Therefore, this study can contribute to reducing the damage resulting from landslides by establishing alarm standards of hourly and duration rainfall amount that consider regional characteristics.

## Research procedure

**Figure 1:**
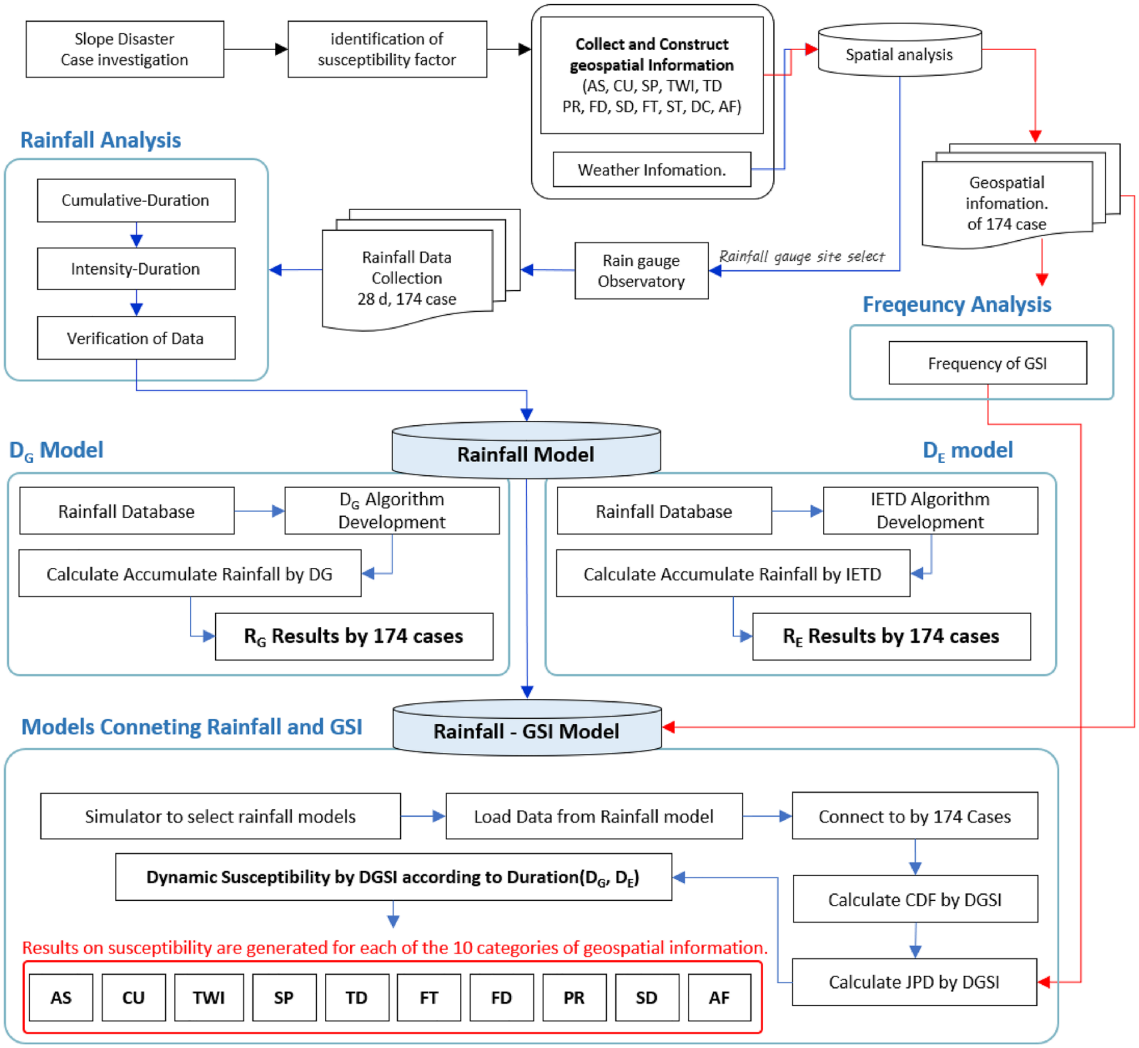
Research methodology flow chart of this study.

Figure 1 shows the research methodology flowchart of this study. We collected landslide cases have occurrence time for dynamic landslide susceptibility analysis and constructed the spatial information, analysed the cases, and collected the rainfall data. First, a rainfall model was developed considering the period between the general rainfall model and the rainfall event. Thereafter, a spatial information convergence model was developed. This study was conducted according to a step-by-step procedure that combined rainfall and spatial information.

## Materials and methods

### Data

#### Landslide event data of South Korea

With an increase in torrential and frequent rains due to abnormal weather, the mountainous regions, which constitute approximately 70% of the Republic of Korea, have been adversely affected due to heavy rainfall accompanied by annual typhoons, notably Rusa in 2002 and Mitag in 2019. The precipitation level in 2020 exceeded twice the annual average from late July to mid-August because of heavy rainfall. In Korea, numerous landslides occur due to unusual weather, which includes frequent typhoons, heavy downpours, and long-term rainfalls. Therefore, there’s a scope of landslide cases from 2007 to 2020 in Korea. There is a high frequency of landslide occurrences in the Republic of Korea, and damage has occurred owing to debris flow and shallow slides. In contrast to naturally occurring ground movement, rainfall is the primary cause of landslides in the Republic of Korea.

We focused on collecting the locations (coordinates), dates, and occurrence times as accurately as possible. Referring to Drone images, Google Earth images, and Naver Road Map view, 241 landslide points were verified. Out of initially-collected 241 data points, 174 data points were finalised after excluding the data points where the date and time of occurrence were unknown, and coordinates or rainfall data were missing.

**Figure 2:**
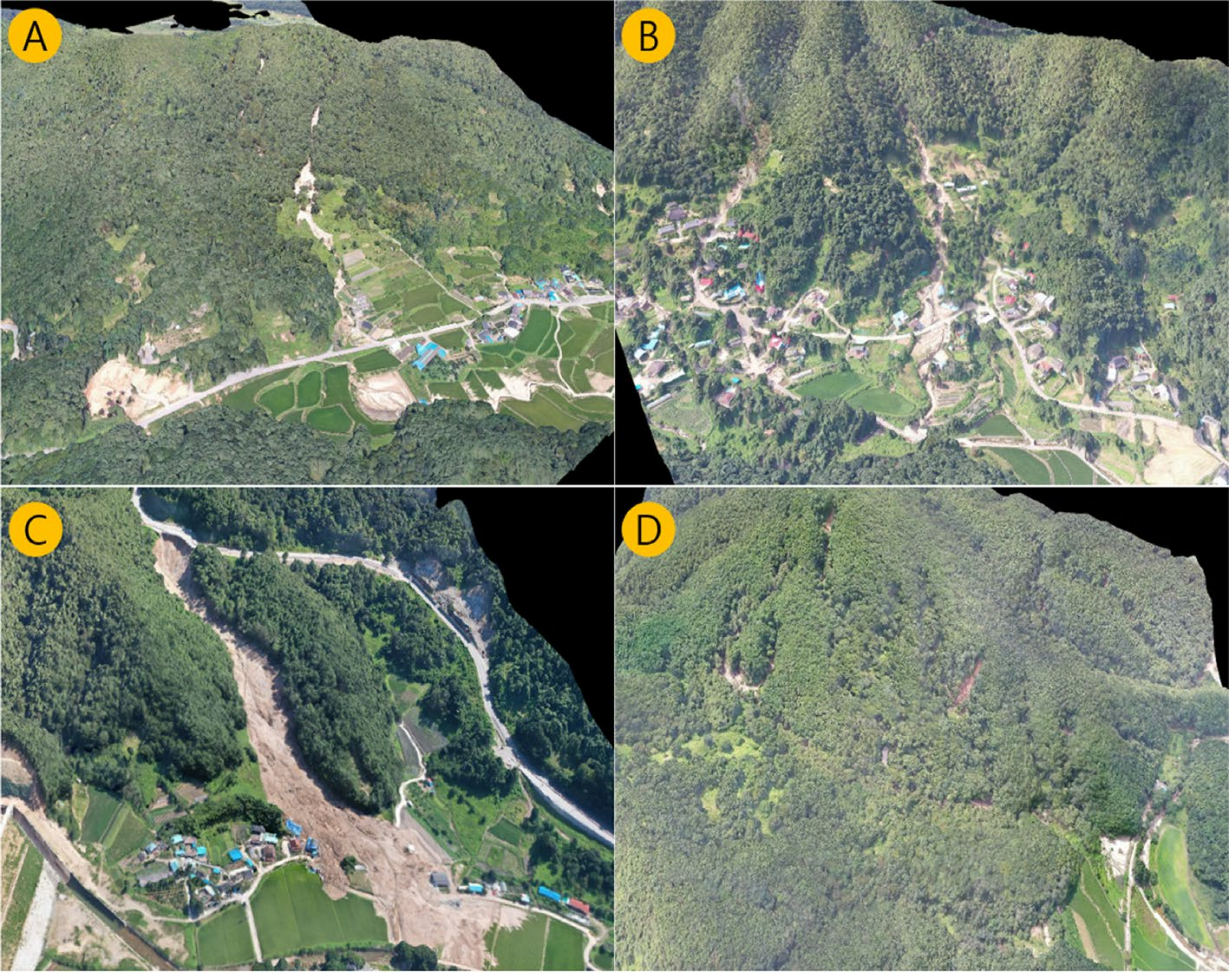
Dronographs of a few significant landslides that occurred at (**A**) Sun-chang, (**B**) Asan, (**C**) Gok-seong, and (**D**) Jang-Su in 2020(created using DJI INSPIRE 2 Drone, PIX4Dmapper and DroneDeploy software, https://www.pix4d.com, www.dronedeploy.com).

The data which was used to create Fig. 2 was sourced from on-site surveys performed using drones in the 2019–2020 landslide areas, National Disaster Management Research Institute reports, Ministry of the Interior and Safety reports, and media surveys. A total of 107 landslide data points were obtained through field surveys during 2019 and 2020. Figure 2 shows some of the drone survey sites to confirm the exact triggering point.

#### Geospatial data

**Figure 3:**
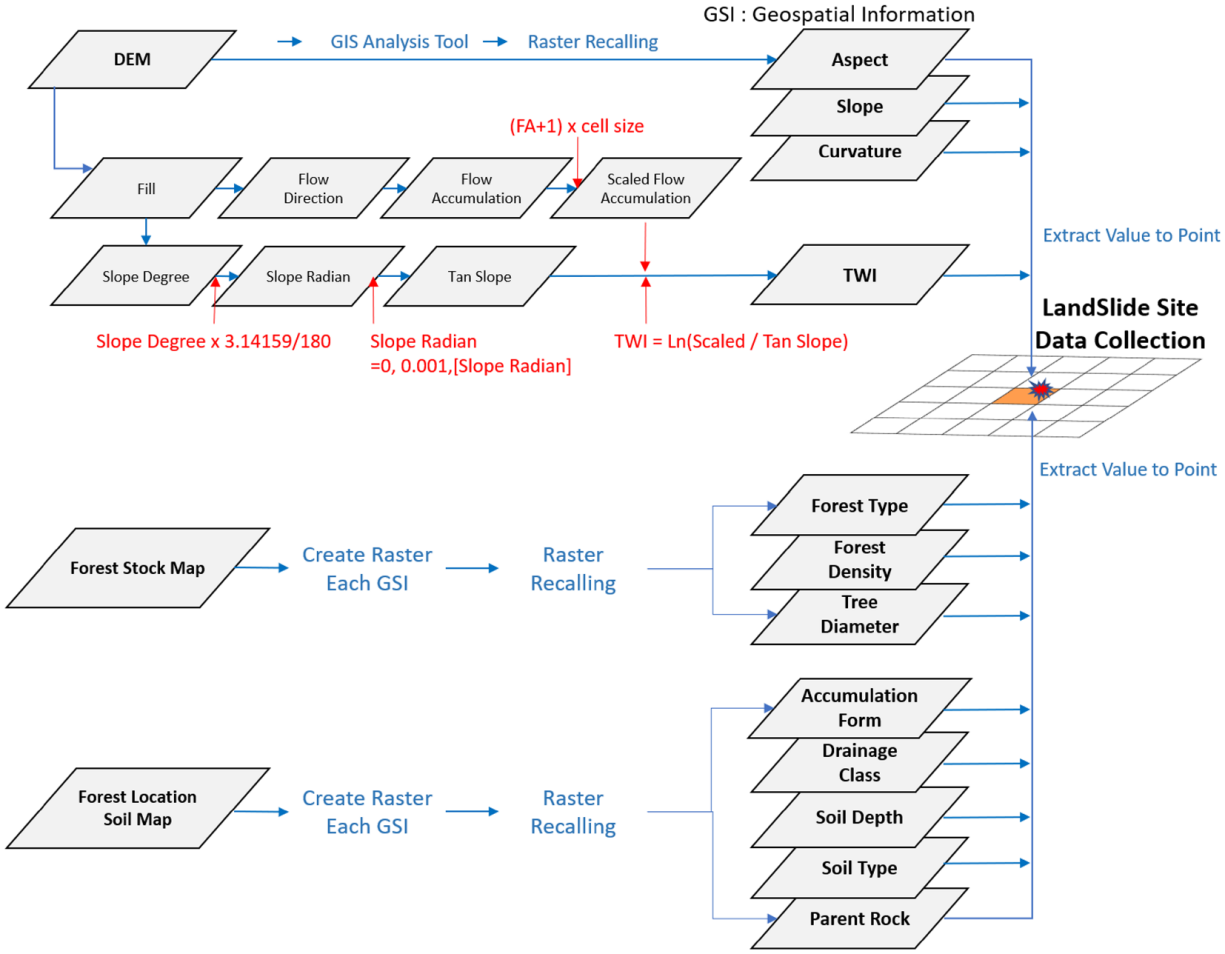
GSI collection procedure of landslide site data.

**Figure 4:**
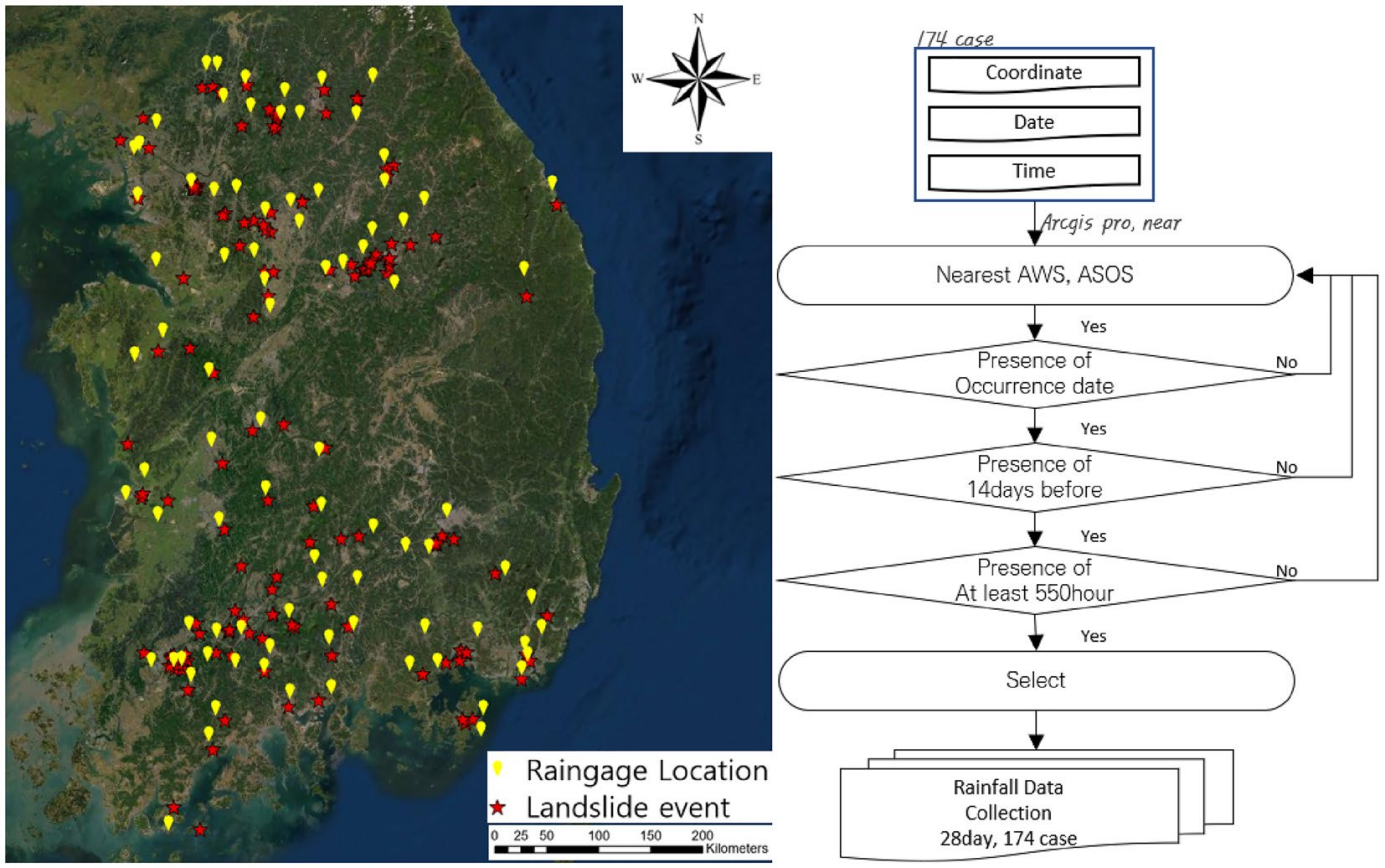
(left) Rain gauge and landslide 174 sites during 2007–2020 in South Korea, (right) rainfall data collection procedure (created using ArcGIS Pro, 2.9.2, https://www.esri.com).

DGSI data were extracted from the GSI built in point data where the collapse occurred. The data utilised spatial information from slopes, aspect, curvature, soil, forests, trees, and drainage ratings used^32–35^. In addition, the parent rock and bedrock lithology was used in landslide analysis^15,36^. Altitude is considered a major factor in landslide analysis but was excluded in this study because we also considered landslide history data in mountainous areas with low altitudes around roads and residential areas. Forest location soil maps and stock maps are vector files that contain a considerable amount of field information. Each GSI was first converted into a raster file and later extracted at the point of landslide occurrence using the GIS analysis tool. The procedure for constructing the data on landslide occurrence is shown in Fig. 3. The data source was a digital elevation model with a 5 M spatial resolution developed based on a 1:5000 map provided by the National Geographic Agency and a forest stock map provided by the Korea Forest Service, which uses a 1:5000 map to utilise clinical types, clinical density, and clinical economy data. The 1:25,000 forest stock map was created through contour modification using a digital aerial photograph and five pieces of information, as shown in Fig. 3. All GSI related to landslide event is constructed to extract the characteristics of event points, as shown in Fig. 4.

#### Rainfall data

Reliable rainfall data are required to confirm the GSI of the collapse point, precipitation, and possibility of collapse and to prepare a risk map. Rainfall data were extracted from the automatic weather system and automated synoptic weather system in Korea. The yellow colour on the left in Fig. 4 indicates the weather system used to extract data. Recently, radar data have been collected to facilitate precise rainfall prediction^37–41^, but the information on landslide occurrence points built in this study used information from 174 points since 2007. Therefore, we had no choice but to rely on past data recorders.

##### Selecting duration

In general, the most frequently used period for rainfall data analysis in landslide disasters is 24 and 48 h. However, 5 and 20 day-periods were used based on the researcher’s judgment^9,43^. Following previous research^43^, 14 days were used to extend the analysis period of the model as much as possible. Therefore, researchers need to make an effort to provide the results from a variety of perspectives by diversifying the rainfall analysis period. This study used 28 days (672 h) of rainfall to confirm that landslides can occur even when the site is exposed to weak rainfall for a long duration.

##### Selecting the station

Ground observation stations for rainfall data collection are operated by the Meteorological Administration. Rainfall data were collected based on the procedure shown on the right side of Fig. 4. This procedure is as follows: First, a ground observation station nearest to the landslide occurrence location was selected for the study. Second, the presence or absence of rainfall data on the date of collapse was checked. Some data points were excluded because there were cases where the data at the time of collapse was not available. Third, we except for the missing hourly rainfall data rate for 14 days (336 h) before the collapse was larger than 5%. Errors may have occurred if missing data were considered in the study because the effective cumulative rainfall cannot be accurately measured as the time between rainfall events is affected. Observation stations were selected, and rainfall data for the site were collected by determining if there was at least 550 h of data from the time of the collapse (Fig. 4). The rain gauge station was geo-coded. The locations and selected points are shown on the left-hand side of Fig. 4. A total of 114 rain gauge stations were selected as the observation stations closest to the 174 collapse case points in this study using a near tool loaded in ArcGIS Pro. However, 26 rain gauge stations were not available due to missing data, discontinued operations, and loss of historical data. Finally, 88 rain gauge stations based on the rainfall-measurement-station selection process on the right-hand side of Fig. 4 were used.

### Methods

#### Random variable

Despite its inherent complexity, disaster prediction has been studied using probability distributions based on past experiences. In probability theory, a random variable is a measurable function from one probability space to another, and its value is determined by the outcome of the trial. Specifically, a random variable can have predictive value for events that have not occurred and is a function that provides the potential results of variables that cannot be accurately determined. If it applies to a countable set, it is called a discrete random variable. If it applies to an uncountable set, it is called a continuous random variable. The domain (*Ω*) of a random variable is its probability space, and the codomain (*Ε*, *ε*) of a random variable is its state space. The random variable *X*:*Ω* → *Ε* is derived in state space *E*. This represents the probability that *X* has a value *S*, and the equations are as follows:

1 $${\varvec{P}}\left({\varvec{X}}\in {\varvec{S}}\right)=\boldsymbol{ }{\varvec{P}}\left({{\varvec{X}}}^{-1}\left({\varvec{S}}\right)\right),$$

2 $${{\varvec{X}}}^{-1}\left({\varvec{S}}\right)={\varvec{\omega}}\in \boldsymbol{\Omega}:{\varvec{X}}\left({\varvec{\omega}}\right)\in {\varvec{S}}.$$

#### Probability mass function

The probability mass function (PMF) represents the probability that a random variable has a specific discrete value, whereas the probability density function represents the probability of a random variable assuming continuous values. When variable X:S → R is a discrete random variable determined by sample space S, the PMF is expressed as Eqs. (3) and (4), given below. Here, *x*_*n*_ is the number of cases in the sample space. If the random variable is X, the PMF corresponding to the random variable is expressed by Eqs. (3) and (4):

3 $${{\varvec{P}}}_{{\varvec{X}}}\left({x}\right):{\varvec{R}}\to \left[{{x}}_{1},{{x}}_{2}\cdots {{x}}_{{\varvec{n}}}\right],$$

4 $${{\varvec{P}}}_{{\varvec{X}}}\left({\varvec{x}}\right)={{\varvec{P}}}_{{\varvec{X}}}\left(\boldsymbol{\rm X}={x}\right)={\varvec{P}}\left[{\varvec{s}}\in {\varvec{S}}:{\varvec{X}}\left({\varvec{s}}\right)={x}\right].$$

#### Cummulative distribution function

The cumulative distribution function (CDF) represents the probability that a given random variable is less than or equal to a specific value. The probability is obtained according to the given value of the function and random variable X, and the accumulated graph is called the CDF. The CDF is defined by Eq. (5). The CDF of the discrete random variable *X* with a probability distribution f(x) is denoted by *F*_*X*_(*x*).

5 $${{\varvec{F}}}_{{\varvec{X}}}\left({\varvec{x}}\right)={\varvec{P}}\left({\varvec{X}}\le {\varvec{x}}\right)=\sum_{{\varvec{t}}\le {\varvec{x}}}{\varvec{f}}({\varvec{t}}).$$

This represents the cumulative probability of *x*, which denotes any one of the accidents. In the CDF operation, the condition in Eq. (6) holds for any value *x* of random variable X, and if the two random variables are such that a < b, Eq. (7) must be used instead.

6 $${\varvec{P}}\left({\varvec{X}}>{\varvec{x}}\right)=1-{\varvec{F}}\left({\varvec{X}}\right),$$

7 $${\varvec{P}}\left({\varvec{a}}<{\varvec{x}}<{\varvec{b}}\right)={\varvec{F}}\left({\varvec{b}}\right)-{\varvec{F}}\left({\varvec{a}}\right).$$

The CDF has a lower limit value of zero and an upper limit value of 1. Specifically, the CDF represents an accumulation of probabilities, as shown in Eq. (8).

8 $${{\varvec{F}}}_{{\varvec{X}}}\left(-\boldsymbol{\infty }\right)=0,\boldsymbol{ }{{\varvec{F}}}_{{\varvec{X}}}\left(\boldsymbol{\infty }\right)=1.$$

As shown in Eq. (9), CDF, being a right-up function, accumulates probability as the *x* value increases and always either has a positive or zero value.

9 $$\forall {{\varvec{x}}}_{{\varvec{n}}+1}\ge {{\varvec{x}}}_{{\varvec{n}}},\boldsymbol{ }{\boldsymbol{ }{\varvec{F}}}_{{\varvec{X}}}\left({{\varvec{x}}}_{{\varvec{n}}+1}\right)\ge {{\varvec{F}}}_{{\varvec{X}}}\left({{\varvec{x}}}_{{\varvec{n}}}\right).$$

The discrete random variable can be identified using the PMF in Eq. (10) using the CDF value, where ε denotes the value of the difference.

10 $${{\varvec{x}}}_{{\varvec{i}}}\in {{\varvec{S}}}_{{\varvec{X}}},\boldsymbol{ }{{\varvec{F}}}_{{\varvec{X}}}\left({{\varvec{x}}}_{{\varvec{i}}}\right)-{{\varvec{F}}}_{{\varvec{X}}}\left({{\varvec{x}}}_{{\varvec{i}}}-{\varvec{\varepsilon}}\right)={{\varvec{P}}}_{{\varvec{X}}}\left({{\varvec{x}}}_{{\varvec{i}}}\right).$$

#### Joint probability distribution

A bivariate distribution refers to the joint probability distribution (JPD) of random variables. It is mainly used for weight analysis. It is expressed by Eqs. (11) and (12), and the sum of the coupling probabilities for two discrete random variables *X* and *Y* can be expressed as shown in Eq. (12).

11 $${{\varvec{P}}}_{{\varvec{X}},{\varvec{Y}}}\left({\varvec{x}},{\varvec{y}}\right)={\varvec{P}}\left({\varvec{X}}={\varvec{x}},{\varvec{Y}}={\varvec{y}}\right),$$

12 $$\sum_{{\varvec{i}}}\sum_{{\varvec{j}}}{\varvec{P}}\left({\varvec{X}}={{\varvec{x}}}_{{\varvec{i}}},{\varvec{Y}}={{\varvec{y}}}_{{\varvec{j}}}\right)=1.$$

When using the JPD, each occurrence frequency can be considered for each DGSI, which is the *X* value, and *Y* attempts to reflect the changed value of rainfall over time.

#### System dynamics model

System dynamics is a theory dealing with interactions between entities in dynamic systems and presenting concepts for system dynamics construction^44,45^. Powersim is an analytical design tool for reproducing system dynamics, and it can perform a simulation function that automatically converts rapidly changing input data to provide the desired output. System dynamics can set a range (time dependent like rainfall duration) to derive results by setting not only the relationship between single variables but also between several variables. The mathematical expression of the state in which the system state change occurs is Eq. (13), and the time variable is expressed as given in Eq. (14) so that the process of changing the time and variables can be calculated in a continuous and procedural order.

13 $$New\;States={\int }_{t=1}^{t=n}({\int }_{t=1}^{t=n}Variation1)dt)dt),$$

14 $${F}_{X}={\int }_{t=1}^{t=n}\left(\frac{F\left({x}_{1+\Delta t}\right)-F\left({x}_{t}\right)}{\left(t+\Delta t\right)-t}\right)dt={\int }_{t=1}^{t=n}f\left({x}_{t}\right)dt.$$

#### Model design principle

**Figure 5:**
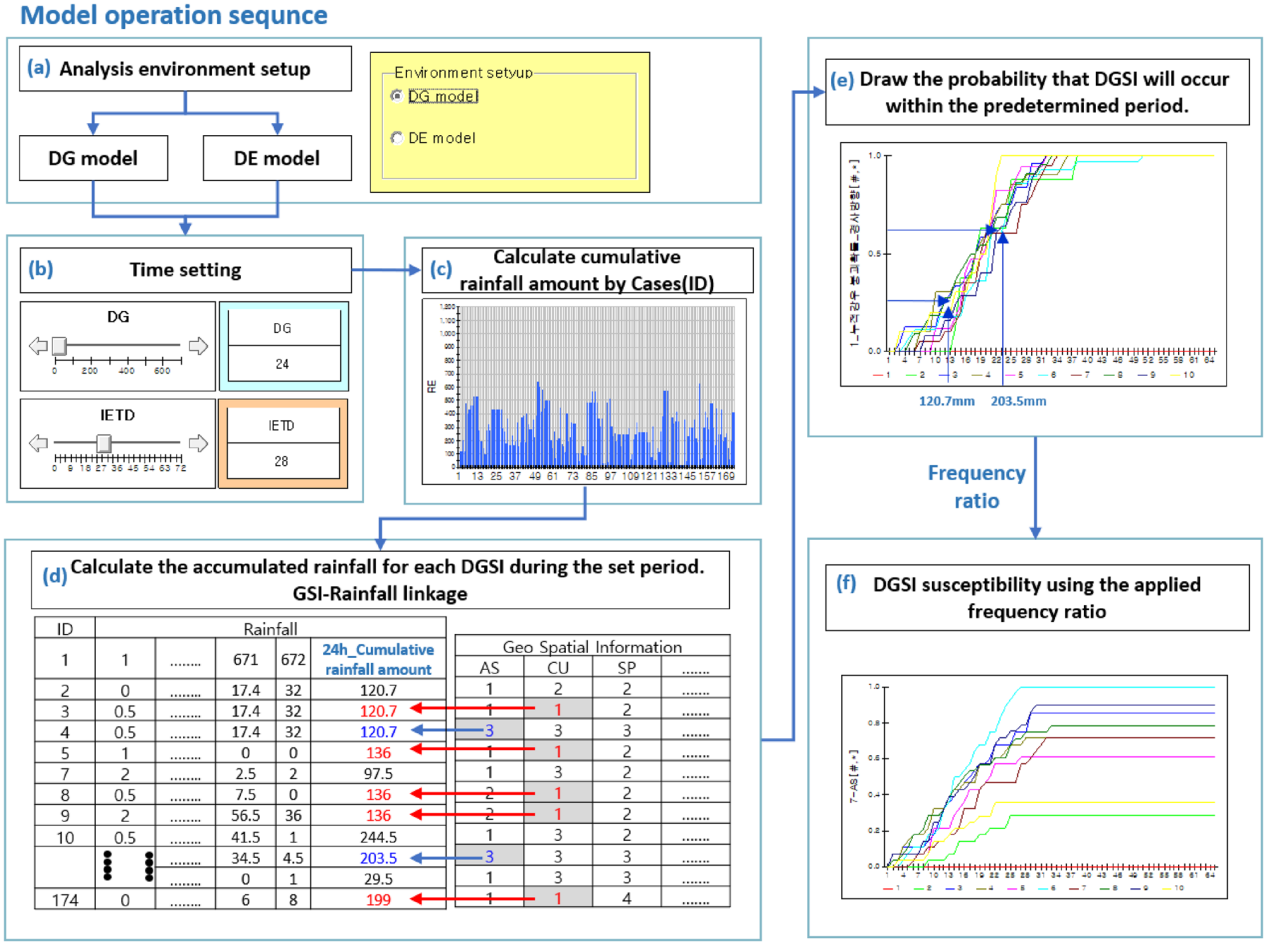
Model operation sequence and principle: (**a**) environmental setup, (**b**) time setting, (**c**) calculate cumulative rainfall, (**d**) calculate method of susceptibility, (**e**) result of susceptibility, and (**f**) the result applied occurrence frequency by DGSI.

The model operation steps are depicted in succession in Fig. 5. As shown in Fig. 5a, the users select an option from the general rainfall duration (D_G_) and the effective rainfall duration (D_E_), which is calculated using the IETD time. Then, the users can select the rainfall period they want to investigate (Fig. 5b). If the user does not take into account the dry period, users may choose D_G_ to input the times from the beginning of the rain to the present. Alternatively, the user can choose the preferred IETD time if it is assumed that effective rainfall influences the probability of landslides. Then the accumulated rainfall is calculated for each case during the selected period (Fig. 5c). The accumulated rainfall results of 174 cases are matched by DGSI grade in Fig. 5d to the accumulated rainfall that resulted in the landslide. For all DGSI factors, the cumulative distribution function converges to 1. Because the low-frequency area might have higher susceptibility at the same cumulative rainfall, the random variable outcomes of the high-frequency and low-frequency DGSI could not be reflected properly. As a consequence, the final random variable in Fig. 5f was derived by applying the frequency of occurrence results analysed by DGSI.

## Design model

### Construction of dataset

**Figure 6:**
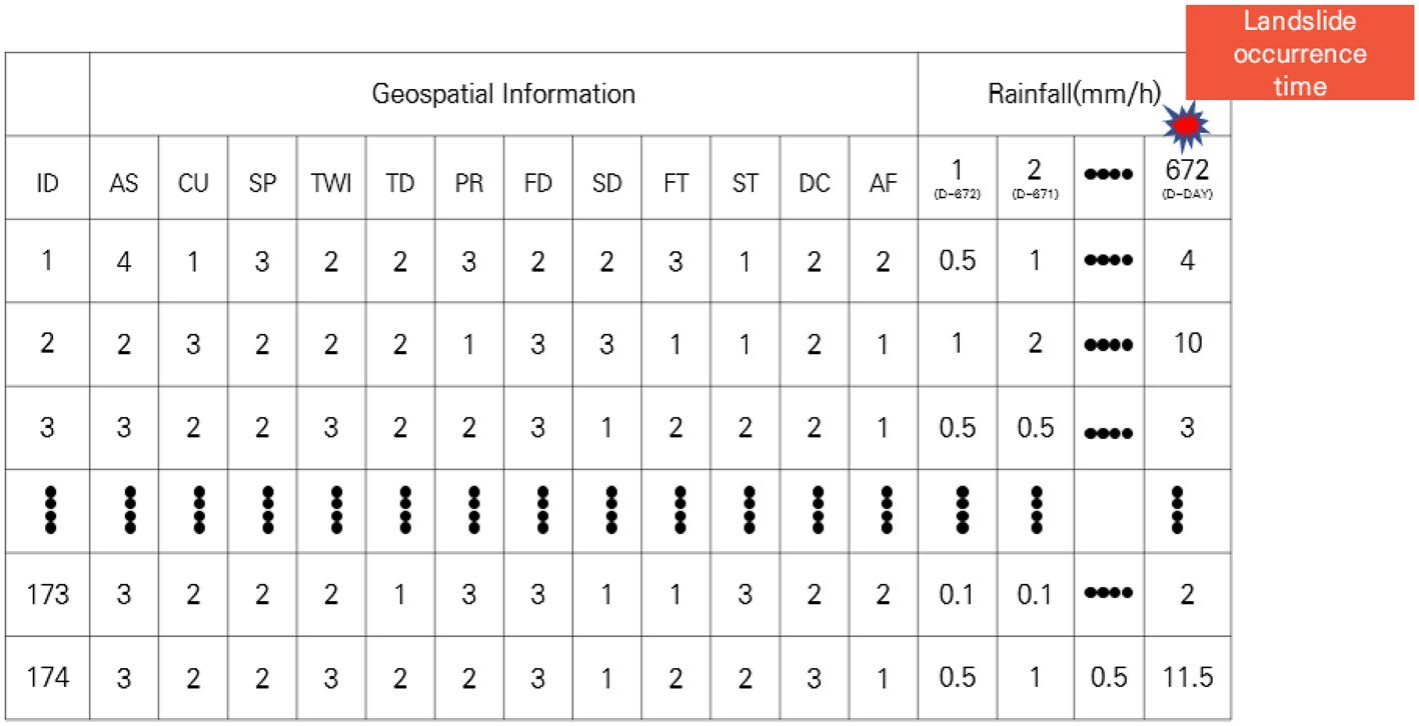
Comprehensive data for dynamic susceptibility analysis. [Aspect (AS), curvature (CU), slope (SP), TWI, tree diameter (TD), parent rock (PR), forest density (FD), soil depth (SD), forest type (FT), soil type (ST), drainage class (DC), and accumulated form (AF)].

The comprehensive data for the dynamic susceptibility analysis of landslides comprised the case number (ID), GSI, and hourly rainfall data, as shown in Fig. 6. There exists a total of 174 points in which each point has its own GSI and 672 h of rainfall data. The GSI consisted of aspect, curvature, slope, TWI (topographic wetness index), tree diameter, parent rock, forest density, soil depth, forest type, soil type, drainage class, and accumulated form. Rainfall data was composed of data collected over 28 days before the time of occurrence (D-day, 672 h).

### Rainfall model

There exist two configurations of data for analysing the possibility of occurrence of detailed GSI through system dynamics modelling. One is the graded GSI at the accident site, and the other is the previous hourly rainfall data recorded for 672 h from the time of the collapse.

**Figure 7:**
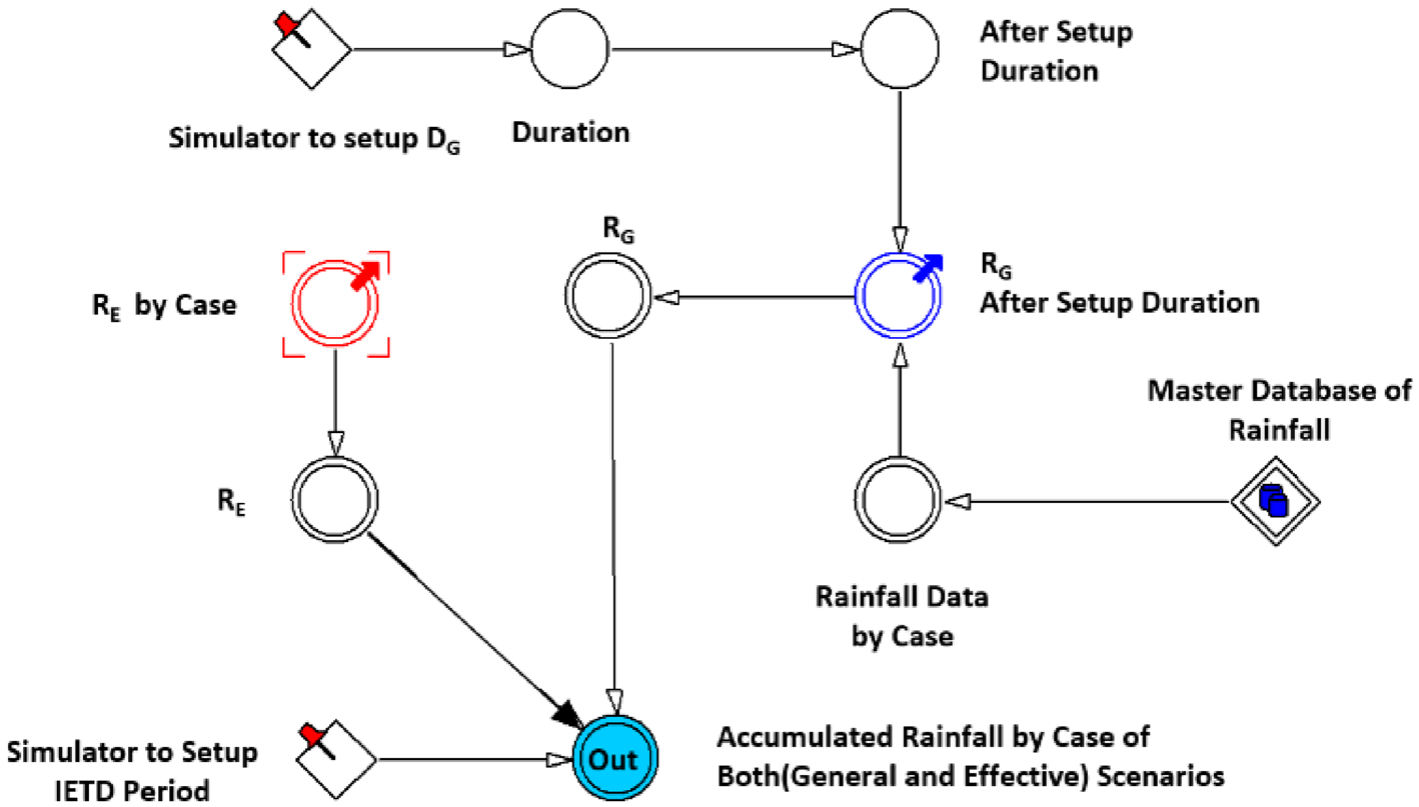
Flow diagram of D_G_ and D_E_ in the rainfall model.

**Figure 8:**
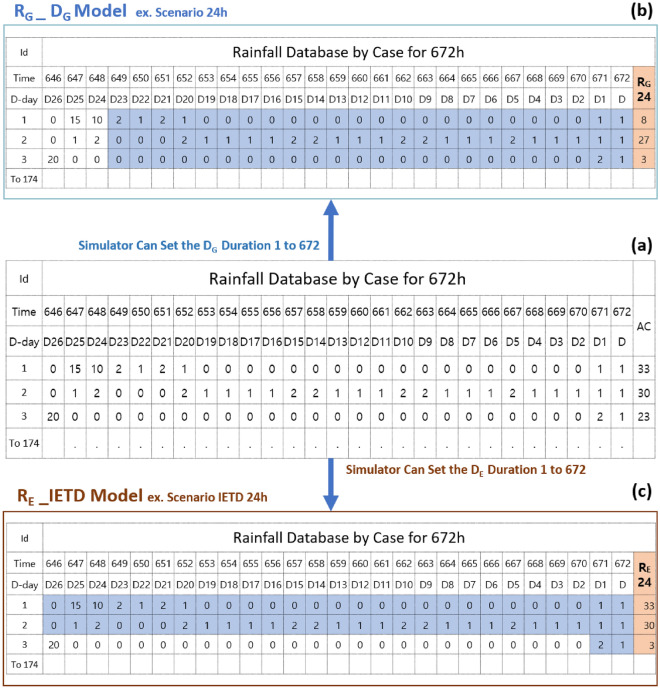
Schematic diagram used for deciding R_G_ and R_E_ values: (**a**) collected rainfall data (**b**) the calculating method in R_G,_ (**c**) the calculating method in R_E_.

First, only the rainfall data were processed. Output data according to the rainfall scenario (period setting) were determined by the cumulative rainfall time setting simulator () combined with the constant variable (◇), which indicates a 672-h rainfall and is determined through the functional expression of the auxiliary variable (○). The rainfall model sets the cumulative rainfall time *D*_*G*_ in two ways to derive the general cumulative rainfall (*R*_*G*_). Next, the effective cumulative rainfall (*R*_*E*_) result is derived by setting the time between rainfall events (IETD). Figure 7 shows a flow diagram of a powersim that can calculate rainfall amounts to link GSI before susceptibility analysis. Figure 8 shows a schematic of the difference between Fig. 8b in the case of *R*_*G*_ and Fig. 8c in the case of *R*_*E*_ values. It can be observed that cumulative rainfall is derived by recognising the data shaded in blue, and significantly different values can be obtained according to the arrangement of the rainfall data. Figure 8a shows the collected rainfall data, (b) shows the result of setting 24 h as a period, and (c) shows the results of the IETD model.

#### D_G_ analysis model design

**Figure 9:**
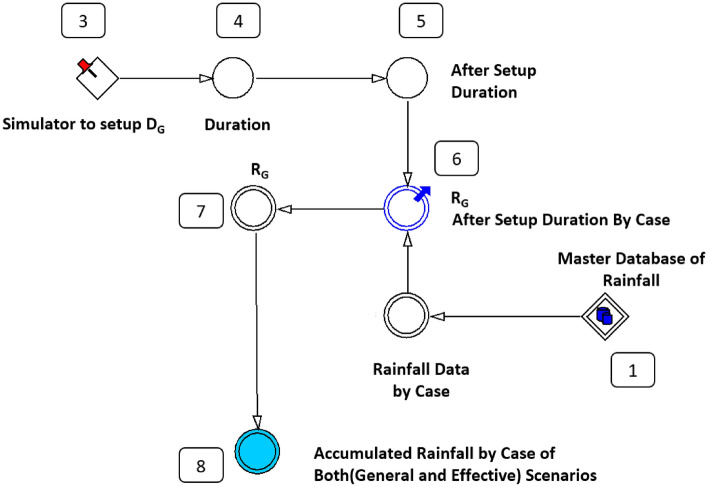
Flow diagram for determining R_G_ by setted D_G_.

The R_G_ extraction model undergoes an eight-step state change process. The roles of (1) to (8), as shown in Fig. 9, are as follows. In (1), the amount of rainfall per case is recognised from the rainfall master data. In (2), the data are sorted. (3) acts as a simulator to select the *D*_*G*_. In (4), the selected *D*_*G*_ is recognised. In (5), we recognise the set period. In (6), the general cumulative rainfall for each case is calculated. In (7), a range to be output is determined, and in (8), a conditional classification is performed to determine the analysis environment. Figure 9 shows the flow for the operation of the general cumulative rainfall model, and the functions used in each step are included in Eq. (A.1). Parts of calculated rainfall results are included in Eq. (A.4).

#### D_E_ analysis model design

**Figure 10:**
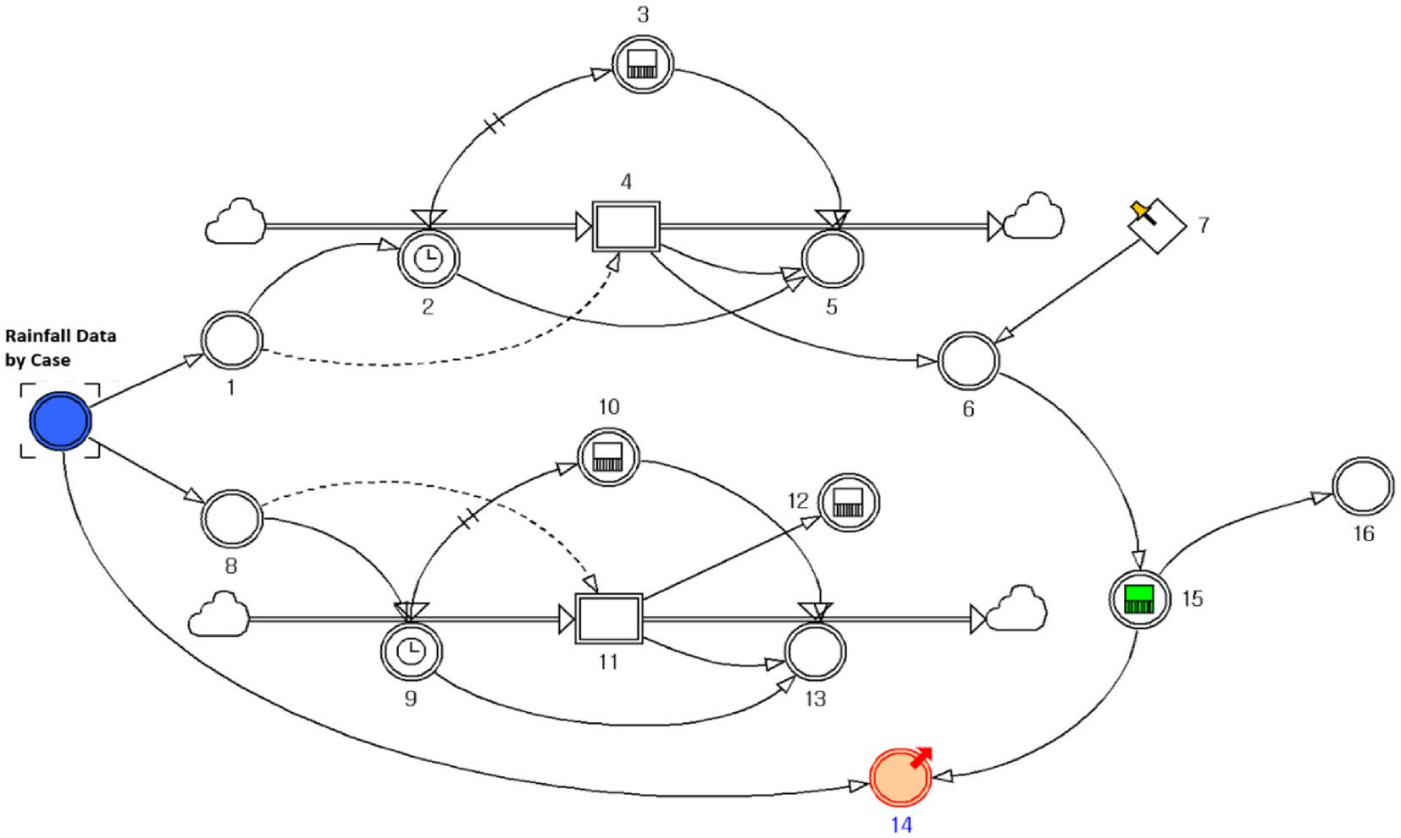
Flow diagram for determining R_E_ by setted D_E_ (IETD).

D_E_ is the rainfall duration, which is included in calculating the cumulative rainfall determined by the IETD. D_E_ modelling was designed to derive the effective cumulative rainfall (R_E_), excluding the rainfall-free period, by setting the time between rainfall events. Figure 10 shows the model design for deriving the effective cumulative rainfall and is used as input data for *R*_*E,*_ depending on the case. To summarise the order of the effective cumulative rainfall analysis model, the non-rainfall period (TN) and rainfall period for each point were calculated. Then the integrated value was derived for 174 cases after the last period in which the cumulative rainfall time exceeded IETD*. R*_*E,i*_ is the effective cumulative rainfall in case *i*, and *R*_*t*_ is the hourly rainfall. *t* = *last* is the last time when TN minus IETD is greater than zero, and *t* = *event* denotes the time until the accident occurs.

15 $${R}_{E,i}={\int }_{t=last}^{t=event}{R}_{t}dt.$$

The *D*_*E*_'s decision requires a procedure to count non-rainfall periods and valid rainfall periods for each case. Figure 10 shows a rainfall model that determines the *R*_*E*_ according to the IETD time determination, and the calculation is performed according to the R_E_ determination method provided in the bottom table in Fig. 8, which uses the function given in Eq. (A.2). Parts of calculated rainfall results are included in Eq. (A.5). R_G_ was applied differently to each case in which D_E_ and accumulated rainfall were influenced by the IETD setting for each case built in this study.

#### GSI-rainfall linkage model design

**Figure 11:**
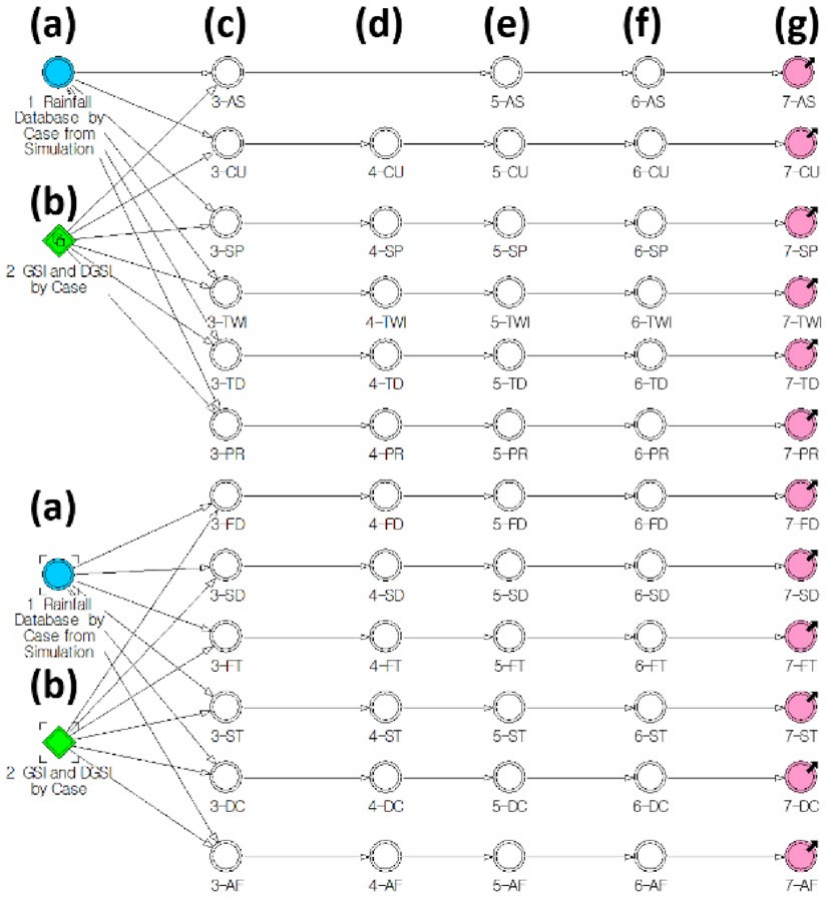
Flow diagram for fusion of GSI and rainfall results (**a**) input data (accumulated rainfall from D_G_, D_E_ model), (**b**) input data (geospatial information of 174 location), (**c**) ~ (**g**) function to draw susceptibility refer to A.3.

The logic behind the rainfall-geospatial information model is shown in Fig. 11. Figure 11a was used as an *R*_*G*_ or *R*_*E*_ data input variable, which is the rainfall simulation result. Figure 11b is a constant variable that plays a role in reading the first established GSI from MS Excel. Figure 11c recognises the detailed GSI for each case and plays a role in arranging the cumulative rainfall in the corresponding case. Figure 11d sorts and checks some data, and Fig. 11e checks the cumulative amount of DGSI as the cumulative rainfall increases for each case. Figure 11f provides the results of calculating the probability according to each rainfall event by DGSI grade. Figure 11g represents the step for calculating the cumulative probability, and the result thus obtained is provided for each DGSI using the results of Number 6 derived for each rainfall event. The design of the dynamic system for checking the dynamic susceptibility of each detailed GSI consists of processes, such as conditions, classification, arrangement, application, variable definition, cumulative sum, and result output. The roles and functions for each number described above are included in Eq. (A.3). The parameters within each system were extracted as array data with detailed values for the amount of GSI in each processing stage for 174 cases and 672 h. Finally, all results of the model were converted into Excel data. This approach has the advantage of being able to geocode and use GIS field data; therefore, it can effectively express visual outputs. In addition, data conversion is convenient; therefore, it is possible to continuously update the results by including landslide disaster cases.

## Results

### Frequency ratio (FR) analysis for weight of DGSI

In this study, grading refers to the conversion of categorical data into a format that can be used for statistical analyses. In this paper, tree diameter, parent rock, forest density, forest type, soil types, drainage class, and accumulated form at the time of constructing spatial information were classified and utilized according to definitions because they are categorical spatial information data^46^. However, in the case of continuous variables, classes were classified based on the principle of equal distribution, referring to previous studies. Aspects are generally classified into nine classes^12,29^, curvature is sometimes classified into five classes^46^, and concave, straight, convex are sometimes used for analysis by classifying them into three classes^13,47,48^. The slope was classified into five classes^49^. In the case of TWI, three^13,49^, five^29,47,48^, and six^50^ types were used under the analysis and judgment of the researcher. In the case of soil depth, five categories were used^47,48^. Table 1 shows the frequency (F) of each DGSI where the landslide occurred. The analysed frequency was linked to each rainfall according to changes in general cumulative rainfall and effective cumulative rainfall and was used as a weight when analysing the results of the susceptibility of each DGSI due to rainfall-spatial information combination modelling.

**Table 1:** Range of values for each grade and FR of DGSI.

GSI	DGSI (grade)	Classification value	F	FR (%)
Aspect (AS)	1	x < 0	0	0
	2	0 ≦ x < 22.5	8	4.6
	3	22.5 ≦ x < 67.5	24	13.8
	4	67.5 ≦ x < 112.5	20	11.5
	5	112.5 ≦ x < 157.5	17	9.8
	6	157.5 ≦ x < 202.5	28	16.1
	7	202.5 ≦ x < 247.5	20	11.5
	8	247.5 ≦ x < 292.5	22	12.6
	9	292.5 ≦ x < 337.5	25	14.4
	10	337.5 ≦ x < 360	10	5.7
Curvature (CU)	1	–	80	46.0
	2	0	0	0
	3	+	94	54.0
Slope (SP)	1	x < 15	24	13.8
	2	x < 30	95	54.6
	3	x < 45	50	28.7
	4	x < 60	5	2.9
	5	60 ≦ x	0	0
TWI	1	x < 1.5	0	0
	2	x < 3	32	18.4
	3	x < 4.5	87	50.0
	4	x < 6	41	23.6
	5	x < 7.5	8	4.6
	6	x < 9	2	1.1
	7	x < 10.5	1	0.6
	8	x < 12	1	0.6
	9	x < 13.5	2	1.1
	10	13.5 ≦ x	0	0
Tree diameter (TD)	1	None or rare	15	8.6
	2	Small wood	34	19.5
	3	Middle wood	119	68.4
	4	Large wood	6	3.5
Parent rock (PR)	1	None	0	0
	2	Igneous rock	93	53.5
	3	Sedimentary rock	22	12.6
	4	Metamorphic rock	59	33.9
Forest density (FD)	1	None	7	4.0
	2	Low	8	4.6
	3	Medium	9	5.2
	4	High	150	86.2
Soil depth (SD)	1	0	0	0
	2	x < 10	6	3.4
	3	x < 20	43	24.7
	4	x < 30	116	66.7
	5	30 ≦ x	9	5.2
Forest type (FT)	1	None	1	0.5
	2	Coniferous forest	73	42.0
	3	Broadleaf forest	80	46.0
	4	Mixed	20	11.5
	5	Bamboo	0	0
Soil type (ST)	1	None	0	0
	2	Sandy loam soil	107	61.6
	3	loam soil	30	17.2
	4	Silt loam soil	20	11.5
	5	Silt clay loam	7	4.0
	6	Sandy clay loam	6	3.4
	7	Loamy sand	4	2.3
Drainage class (DC)	1	None	3	1.7
	2	Poor	0	0
	3	Ordinary	101	58.0
	4	Good	64	36.9
	5	Excellent	6	3.4
Accumulate form (AF)	1	None	7	4.0
	2	Residual soils	61	35.1
	3	Creep	92	52.9
	4	Colluvial Soil	14	8.0

Analysing the frequency analysis results, no difference between the negative and positive values in the case of curvature was observed, and an FR of 54.5% was observed at 16°–30° in the slope. There is a high frequency of occurrence at slopes below 30° because, in the Republic of Korea, the damage occurred owing to debris flow and shallow slide. The topographic wetness index was the most common, with an FR of 50% in the case of the 3rd grade. In terms of the tree diameter, FR was the highest for medium-sized wood. In terms of the parent rock, FR was the highest in the case of igneous rock, and FR was the highest at 86.2% in areas with high forest density. The FR was 66.7% for soil depth in the 4th grade, and it was the highest when the forest type was evenly distributed, except for bamboo groves. The bamboo grove, which was the 5th grade of the forest type, was not deep-rooted from the surface, and the soil layer was weathered. This caused landslides in the bamboo grove area of Jangseong-gun in 2020. It was expected to possess a high FR, but it demonstrated a low FR because only 0.3% of the country’s land area constitutes bamboo grove. In the case of soil type, FR was the highest at 61.5% in sandy loam soil. In the case of the drain class, it was the highest at a rate of 58% for the 2nd grade. The accumulated form exhibited the highest FR (52.9%) during creep.

### Analysis of RG-GSI model

**Figure 12:**
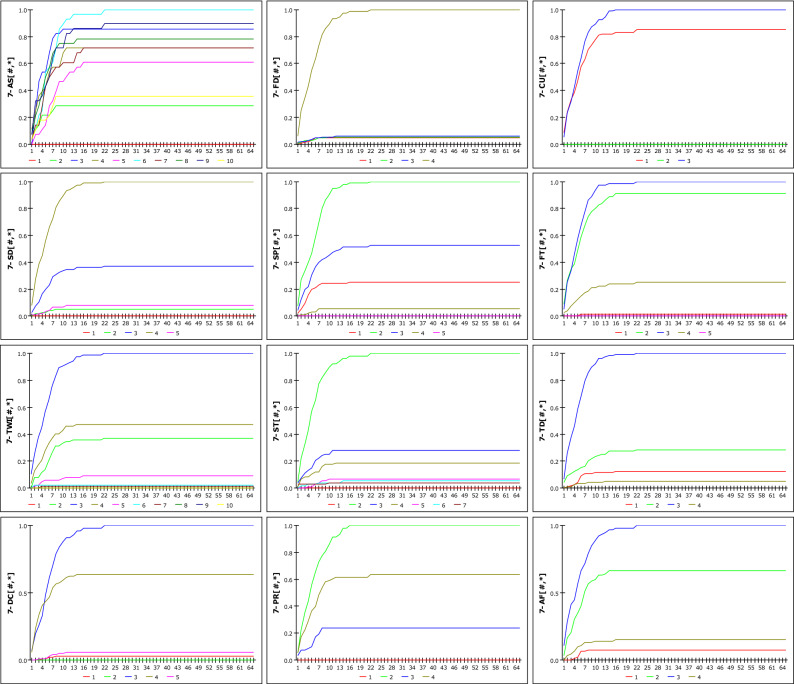
Susceptibility according to cumulative rainfall considering D_G_ = 12 h.

**Figure 13:**
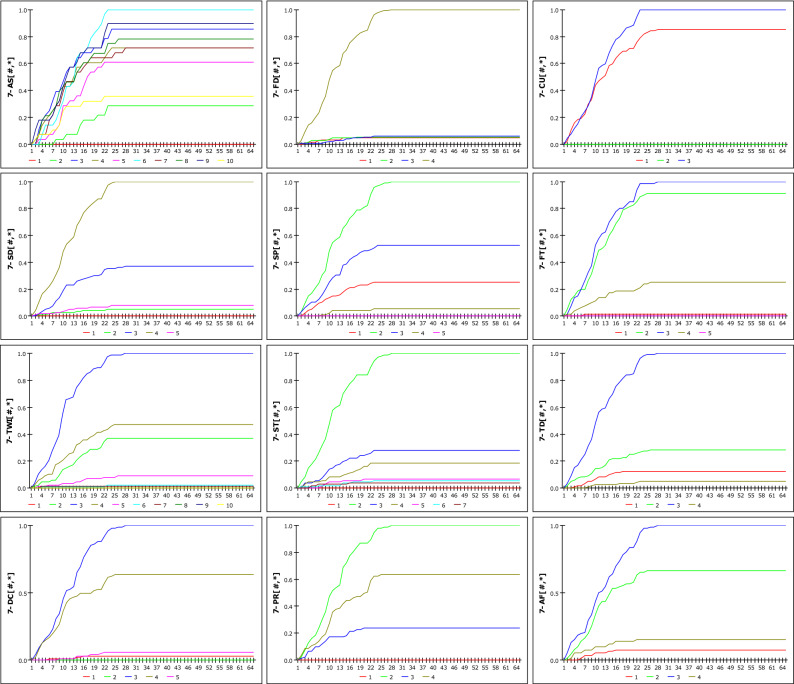
Susceptibility according to cumulative rainfall considering D_G_ = 36 h.

Figure 12 shows the susceptibility according to the increase in rainfall when D_G_ is set to 12 h, as a result of analysing the possibility of occurrence based on R_G_ and DGSI. The susceptibility when D_G_ is set to 36 h is shown in Fig. 13. The two graphs represent 12 DGSI. The vertical and horizontal axes represent susceptibility and cumulative rainfall during the set rainfall period, respectively. One scale on the horizontal axis(X-axis) is divided into 20 mm, and the maximum cumulative rainfall in 672 h is 1300 mm; therefore, it has a scale of up to 65. Consequently, susceptibility is obtained as a discrete result based on changes in rainfall. The reason for the steeper slope at 36 h compared with that at 12 h stems from imperial analysis, which considers that landslides occur due to rainfall over a fixed period. None of the curves approach a probability of one because the weight mentioned in 1 is applied. The results of the dynamic modelling of DGSI considering *D*_*G*_ as 12 h, 24 h, 36 h, 48 h, and 672 h, which are periods that are mainly used as antecedent fall periods, are included the Eq. (A.6).

**Figure 14:**
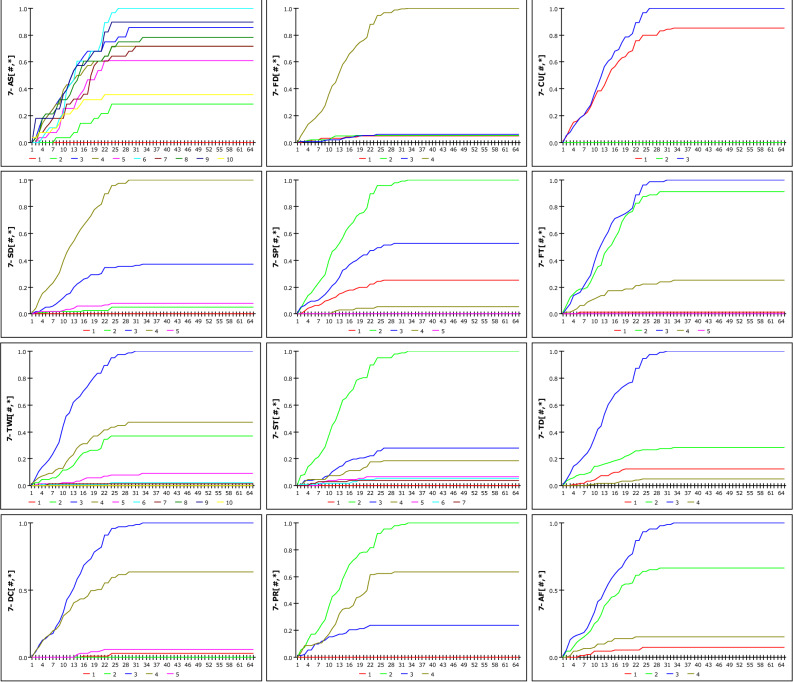
Susceptibility according to cumulative rainfall considering IETD = 12 h.

**Figure 15:**
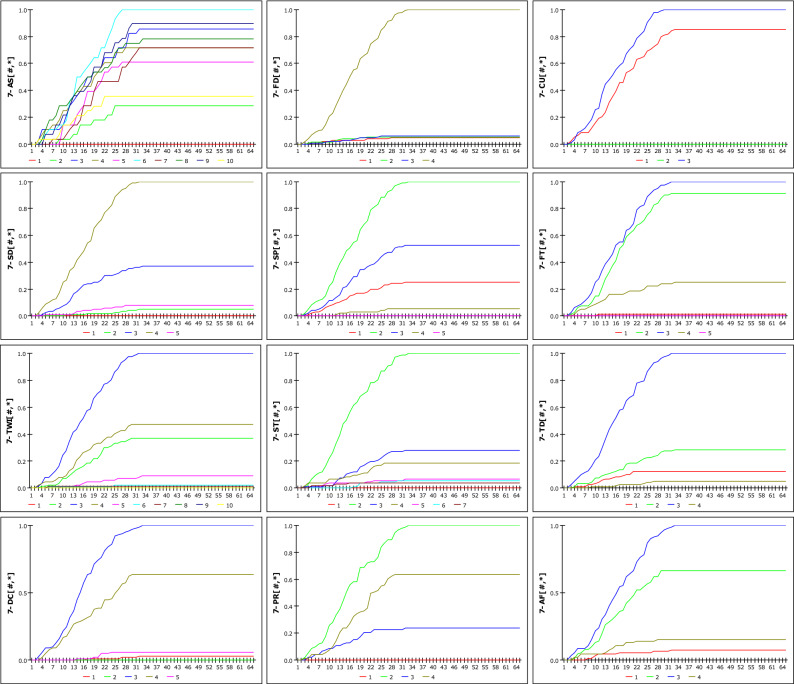
Susceptibility according to cumulative rainfall considering IETD = 36 h.

### Analysis of RE-GSI model

Based on the change in the IETD, we attempted to confirm the susceptibility of landslides to DGSI. The analysis was performed using the same fusion method as in the case of the *R*_*G*_-GSI model, and the *R*_*E*_ results that were derived from the *D*_*E*_ model were used as input data. The results of the susceptibility analysis of *R*_*E*_ and DGSI are shown in Figs. 14 and 15, which represent cases where the IETD was set to 12 h and 36 h, respectively. Unlike the D_G_-GSI model, when the cumulative rainfall approached 560 mm, the susceptibility conversion was 1. The 12-h IETD usually considers an antecedent period of 12 h or more because the subsequent rainfall event is considered when the TN is more than 12 h. Therefore, it can be observed that the susceptibility is lower for the same rainfall than in the case where D_G_ = 12 h. Although it is very difficult to observe regularity owing to the limited number of cases, which is 174 in this study, the analysis results are considered meaningful as a means of finding random variability in the relationship between rainfall characteristics and GSI. In addition, the results of the dynamic modelling for each detailed geomorphological factor considering the DE of 12 h, 24 h, 36 h, 48 h, and 672 h are included in Eq. (A.7).

## Discussion

In order to predict landslides, previous research has gathered rainfall data that triggered landslides. This data has also been utilized to construct the rainfall threshold regression^5,28,43,51,52^ formula and waring criteria^28,43,53,54^ through analyses of rainfall period-rainfall intensity and rainfall period-accumulated rainfall. Previous studies about rainfall thresholds did not allow for dynamic adjustment of the effective rainfall duration and amount since they had been predetermined. The research was performed on combining rainfall data with geospatial information^7,25,26,29,55^, and a system that sent alerts over time^56,57^ was also established. It is challenging to find previous research on a model that simultaneously recognizes and analyses the variables of rainfall period and cumulative rainfall. Therefore, this study developed and applied a methodology that can consider spatial information and rainfall characteristics with complex causal relationships using system dynamics modeling^58^.

We started to estimate the possibility of landslides according to the change in rainfall period and accumulated rainfall for each detailed geospatial information characteristic. The model hypothesizes that landslides are triggered by rainfall events that occurred inside the previous period (defined period by the model) of the occurrence of landslides. The environment with the same rainfall, rainfall time, and geographical information is most likely to face landslides. Therefore, rather than confirming the possibility of landslides by determining the existing D_G_ and IETD in advance through this study, it was determined that the landslide warning system could be developed if the susceptibility could be determined by immediately reflecting the rainfall characteristics that change in real-time. In addition, it was expected to predict the possibility of a landslide at a specific point because spatial information characteristics are reflected.

It is difficult to accurately predict natural landslides. Susceptibility analysis begins with the assumption that they occur under conditions similar to those of past landslides. In addition, it is challenging to derive true values through direct comparison with previous studies because of differences in study target sites, sensitivity derivation methods, analysis factors (GSI and DGSI), and statistical classification techniques^13^. Because natural disasters, such as landslides, floods, and earthquakes, often face difficulty to predict. During the study process, the proposed model offers trustworthy results but also introduces uncertainty^59^. Researchers carried out keeping in mind the uncertainty in landslide prediction^60,61^. Also, despite yielding considerable degrees of accuracy in landslide predictions, the outcomes of different landslide susceptibility models are prone to spatial disagreement; and therefore, uncertainties^23^.

Rather than focusing on deriving accurate results, this study started with the intention of predicting the areas that are more likely to have landslides according to the changing rainfall conditions through new trials.

The model can select a certain collapsed location from among those in the rainfall-affected area when the results of the rainfall are applied. The probability of collapse is larger than in other places if it is a site where several factors with high susceptibility are combined. There were 12 different sorts of listings of geospatial information that were broken down into 66 separate classes. The susceptibility result was calculated for 66 variables in relation to the total accumulation and duration of the rainfall. Since the likelihood of occurrence can be examined for each element, it is simple to comprehend how sensitive an unknown slope made up of several variables is to variations in rainfall. The results are exported as Ms Excel data and can be easily analysed later by linking with spatial visualization programs such as the GIS tool. It was possible to expand the prediction methodology through new attempts of the model. However, dependability issues occasionally surfaced after assessing the accuracy and validity.

**Figure 16:**
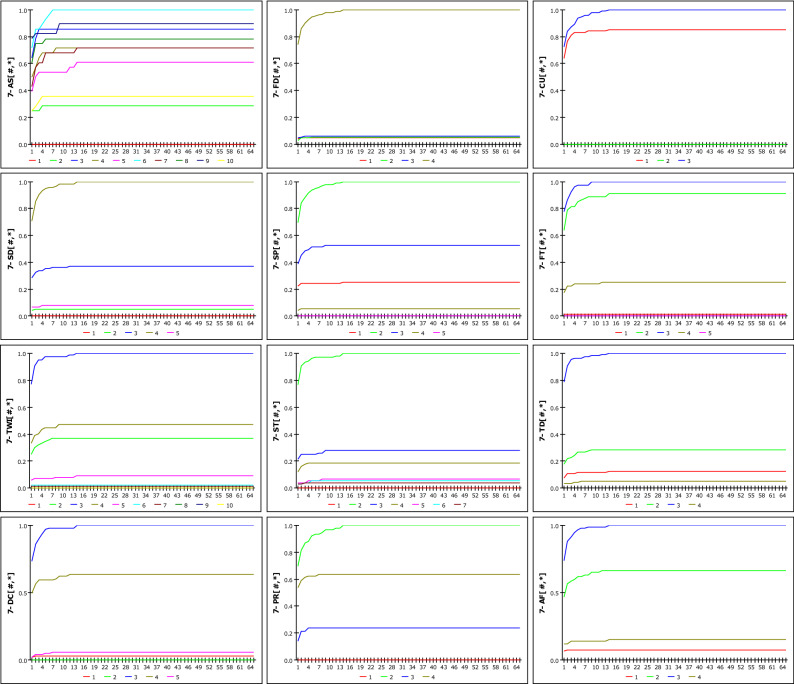
Susceptibility according to cumulative rainfall considering DG = 3 h.

**Figure 17:**
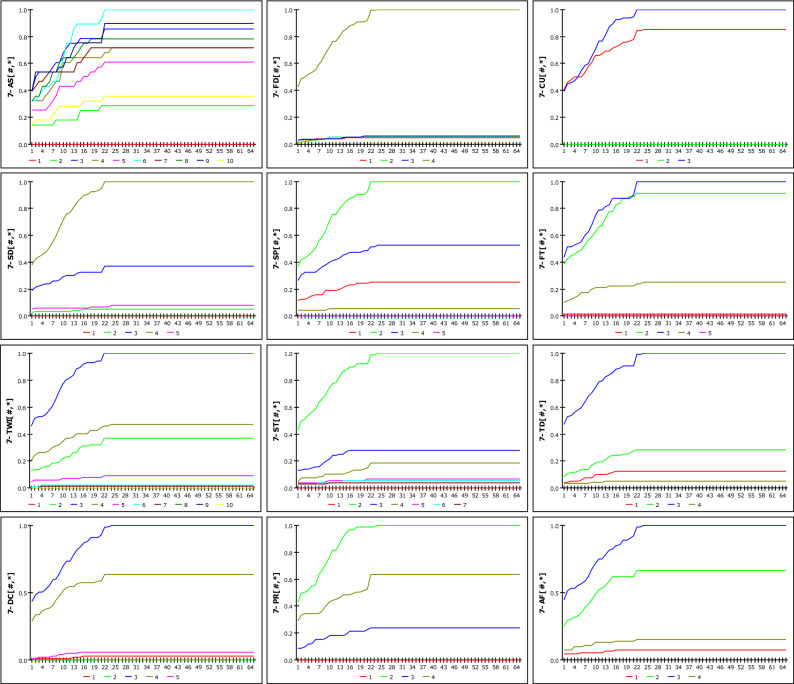
Susceptibility according to cumulative rainfall considering IETD = 3 h.

- In the case of environmental setup, it is necessary to select different DG and DE models according to the weather conditions and period. The DE model (taking into account IETD, which considers the no-rainfall period) will be appropriate in the case of a long rainy season, while the DG model must be taken into account in the case of damage brought on by heavy rain.
- It was proven by checking the rainfall data that the model wasn't appropriate for an extremely short rainfall period. The collapse occurred even though there was no rainfall for 6–8 h before the collapse below, and 2 mm of rainfall occurred for 2 h thereafter. This is because, if the D_G_ is set to 8 h or the IETD is set to 6 h or less, the susceptibility of each DGSI reaches the maximum value at 2 mm of rainfall. This is due to the fact that a cumulative rainfall of 2 mm makes it challenging to determine the occurrence of a landslide. There was no case of collapse due to rainfall only for 3 consecutive hours without preceding rainfall. However, since the model can set a period of 3 h or less, it is inevitably judged that the region has a very high probability of collapse even with a small rainfall, as shown in Figs. 16 and 17. This is considered to be an error of the D_G_ model.

- The model relies on historical landslide-induced rainfall data. Therefore, although there is a possibility that a landslide may occur in the same rainfall and geospatial information circumstance, If the D_G_ is entered for a little time just prior to the landslide, the prediction accuracy may be reduced. This is also because the number of cases is relatively small (174), and it depends a lot on the reliability of the rainfall data at the exact time of occurrence. Because it considers the drying period, the IETD model is frequently used in previous research, and the D_E_ model is also seen to have better results. In addition, since there are cases where there are 10 h of no rainfall in the rainfall data, it is necessary to select an IETD of 10 h or more in order to judge the sensitivity more accurately in the cases collected in this study.
  - Researchers established an effective duration (ED) in advance, which they believed to have had an impact on the occurrence of landslides, and also suggested rainfall thresholds to anticipate landslides observed in the past. For IETD, the 12 h^1^, 24 h^2–8^, 48 h^6,9,10^, and 96 h^6,10^ are predetermined to assess and derive the study outcomes. Additionally, because it utilizes pre-confirmed rainfall data, research that combines geographical information, including studies that perform dynamic analysis, does not allow for the free setting of the time. Based on prior research, the researcher may choose an acceptable period for undertaking research. But because the pattern of rainfall and the features of certain spatial factors are complex, it was concluded that a study on the dynamic analysis approach using this model was required because the abnormal climatic phenomena will increase in the future. The number of cases may be insufficient because gathering accurate data is of the utmost importance, but if big data is established in the future, better outcomes are anticipated.

## Conclusions

Landslides occur due to irregularities in natural phenomena and are difficult to predict. Thus, landslide studies rely on the anticipation of events using rainfall and spatial information characteristics based on historical data. Landslide prediction is due to a complex causal relationship where the characteristics of rainfall, rainfall duration, and spatial information act at the same time. This study tried to establish the relationship with variables by using the system dynamics model useful for analysing complex phenomena and finally to determine the susceptibility of each detailed spatial information according to the change in rainfall period and accumulated rainfall. Data construction and modelling (rainfall modelling design, combine rainfall and spatial information) were performed for susceptibility analysis. The validity of the data is directly related to the accuracy of the prediction. Therefore, in order to secure valid data at the time of construction, rainfall data was collected through a verification procedure. In order to take into account the characteristics of rainfall and spatial information with complicated causal connections, this work devised and utilized a technique employing system dynamics modelling^58^ that is appropriate for complex systems. As a consequence, improvements have been made in the current study that primarily conducts the analysis within the specified rainfall circumstances and individually examines the existing rainfall features and geospatial information. By reflecting the rainfall characteristics that change in real-time, the susceptibility of each spatial information factor can be determined. By introducing a new random variable based on the rainfall duration and cumulative rainfall in a region with complex terrain, combined geology, hydrology, and other factors, it was determined that this research validated the probability of a landslide.

However, this study has some limitations. The data itself is not sufficient because a relatively small number of cases were analysed to obtain accurate rainfall data and occurrence point information. In the future study, more accurate analysis of the susceptibility of the regional unit can be performed using the construction of data at the area unit or the area unit of the collapse occurrence point. In addition, although a new approach has been made with available materials, it is inevitable that accuracy problems arise in predicting natural phenomena. However, the results of this study are expected to contribute to the modification of regional rainfall evacuation standards as reference materials for future landslide warnings. Additionally, they are expected to be used as reference materials for presenting rainfall limits according to hourly rainfall changes by terrain space that has complex geomorphological elements to save lives.

Results may not be precise at this time, but a large amount of information enables it to validate the likelihood of collapse when the duration and amount of rainfall change in real-time. We expect that this research will help to reduce landslide damages by contributing to the landslide warning standards.

## Supplementary Information

Supplementary Information.

## Data Availability

The datasets used and/or analyzed during the current study available from the corresponding author on reasonable request.
