# Supplementary material for: Dynamic landslide susceptibility analysis that combines rainfall period, accumulated rainfall, and geospatial information
Source: Sci Rep. 2022 Nov 1;12:18429. doi: 10.1038/s41598-022-21795-z (PMC9626633; doi:10.1038/s41598-022-21795-z)
Supplement: Supplementary file 1 — Supplementary Information. [file 41598_2022_21795_MOESM1_ESM.docx]

**Eq. (A.1)**

| **No.** | **Role** | **Applied Function** |
| --- | --- | --- |
| ➀ | Input Data | XLDATA("master_DB.xlsx", "DB", "R5C17:R178C688") |
| ② | Array | FOR( i=1..174\| 'Master Database of Rainfall'[i,*]) |
| ③ | Simulator | Simulator |
| ④ | Recognize | 'Duration' |
| ⑤ | Recognize | ‘After Duration’ |
| ⑥ | Array  Condition  Cumulative Sum | FOR( i=1..174\| CUMULATIVESUM ( FOR( j=1..672\| IF(j<(672-'After Setup Duration'), 0, 1)*'Rainfall Data by Case'[i,j]))) |
| ⑦ | Condition | 'RG'[1..174, 672] |
| ⑧ | Condition | IF( 'Setting'=2, 'RG',0)) |

**Eq. (A.2)**

| **No.** | **Role** | **Applied Function** |
| --- | --- | --- |
| ➀ | Conditon | IF('Rainfall of Case'=0, 1, 0) |
| ② | Array,Integral,  Indexing | FOR(i=1..174\|'➀’  [i,INDEX(INTEGER(NUMBER(TIME+1)))]) |
| ③ | Apply | FOR(i=1..174\|DELAYPPL('②’[i], 1, 0)) |
| ④ | Array | FOR(i=1..174\|'➀'[i,1]) |
| ⑤ | Array  Classification | FOR(i=1..174\|IF( ('②'[i]-'③'[i])<0, '④'[i], 0)) |
| ⑥ | Array  Classification | FOR(i=1..174\|IF( '④'[i] >= '⑦', 1, 0)) |
| ⑦ | Simulator | ‘Simulator’ |
| ⑧ | Condition  Classification | IF('Rainfall by Case'>0, 1, 0) |
| ⑨ | Indexing, Integral | FOR(i=1..174\|'⑧'[i,INDEX( INTEGER(NUMBER(TIME+1)))]) |
| ⑩ | Array, Variable define | FOR(i=1..174\|DELAYPPL('⑨'[i], 1, 0)) |
| ⑪ | Array, | FOR(i=1..174\|'⑧'[i,1]) |
| ⑫ | Sum | RUNSUM ( IF( '⑪'=0, 1, 0) ) |
| ⑬ | Array  Classification | FOR(i=1..174\|IF( ('⑨'[i]-'⑩'[i])<0, '⑪'[i], 0)) |
| ⑭ | Array,Sum,  Condition | FOR( i=1..174\|CUMULATIVESUM(FOR( j=1..672\| IF(j<'⑮'[i], 0, 'Rainfall by Case'[i,j])))) |
| ⑮ | Array  runmax | FOR(i=1..174\|RUNMAX(NUMBER  (IF( '⑥'[i]=1, TIME, 0)))) |
| ⑯ | Setting | 672-'⑮' |

| **No.** | **Role** | **Applied Function** |
| --- | --- | --- |
| (a) | Input Data  Condition | IF ('Simulator'=3, 'RE by Case',  IF ('Simulator'=2, 'RG by Case',0))  'RG by Case, RE by Case' |
| (b) | Input Data | XLDATA ("master_DB.xlsx", "Grade_DB", "R5C3:R178C14") |
| (c) | Condition  Array  (ex. Slope) | {FOR (i=1..174\| IF( '2 GSI and DGSI by Case'[i,3]=1, 1, 0)*'1 Rainfall Database by Case from Simulation'[i]),  FOR (i=1..174\| IF( '2 GSI and DGSI by Case'[i,3]=2, 1, 0)*'1 Rainfall Database by Case from Simulation'[i]),  FOR (i=1..174\| IF( '2 GSI and DGSI by Case'[i,3]=3, 1, 0)*'1 Rainfall Database by Case from Simulation'[i]),  FOR (i=1..174\| IF( '2 GSI and DGSI by Case'[i,3]=4, 1, 0)*'1 Rainfall Database by Case from Simulation'[i]),  FOR (i=1..174\| IF( '2 GSI and DGSI by Case'[i,3]=5, 1, 0)*'1 Rainfall Database by Case from Simulation'[i])} |
| (d) | Array | FOR(i=1..174\|'3-SP'[i,1]) |
| (e) | Connect  Condition  Sum  Array  (ex. Slope) | CONCAT ( FOR(i=1..5, j=1..1\| ARRSUM(IF( ('4-SP'[i,*]>0) AND ('4-SP'[i,*]<='Interval Rainfall'[1]), 1, 0))), FOR(i=1..5, j=2..65\| ARRSUM(IF( ('4-SP'[i,*]>0) AND ('4-SP'[i,*]<='Interval Rainfall'[j]) AND ('4-SP'[i,*]>'Interval Rainfall'[j-1]),1, 0)))) |
| (f) | Array  Sum | FOR (i=1..5, j=1..65\| '5-SP'[i,j]  DIVZ0 ARRSUM('5-SP'[i,*])) |
| (g) | Array  Sum | FOR (i=1..5\| CUMULATIVESUM('6-SP'[i,*])) |

**Eq. (A.3)**

**Eq. (A.4)**


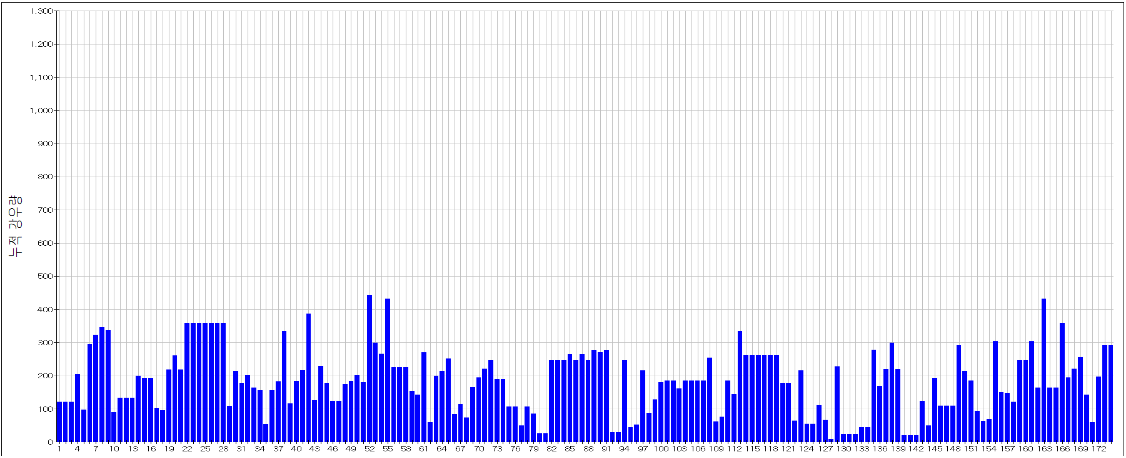


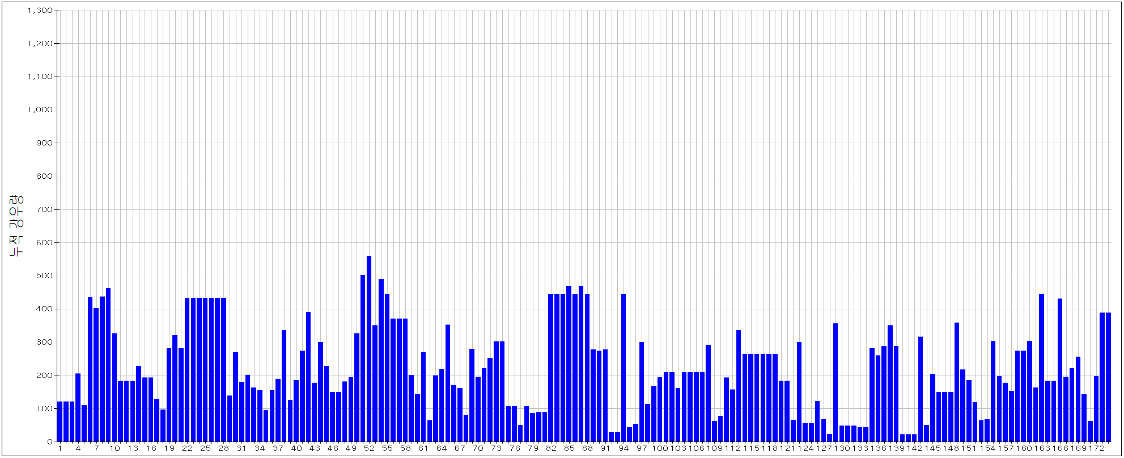


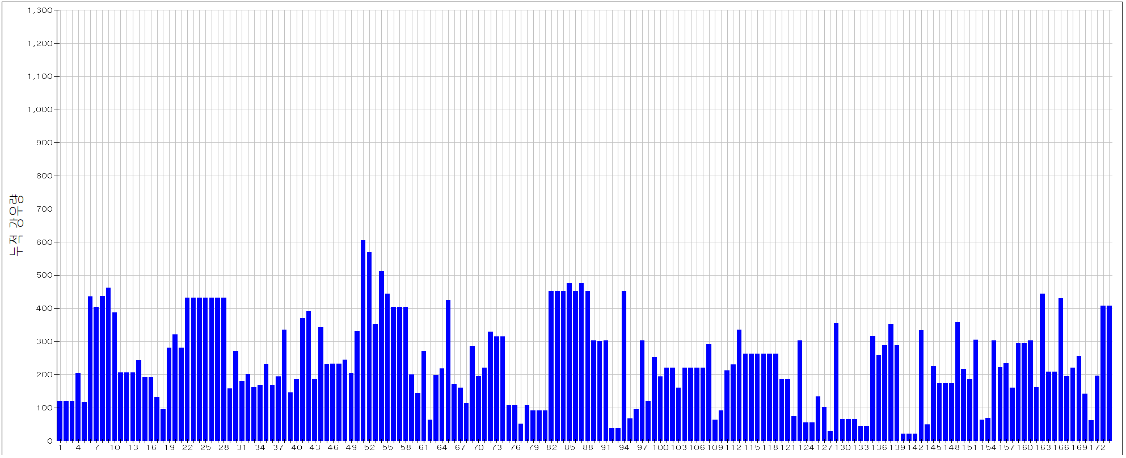

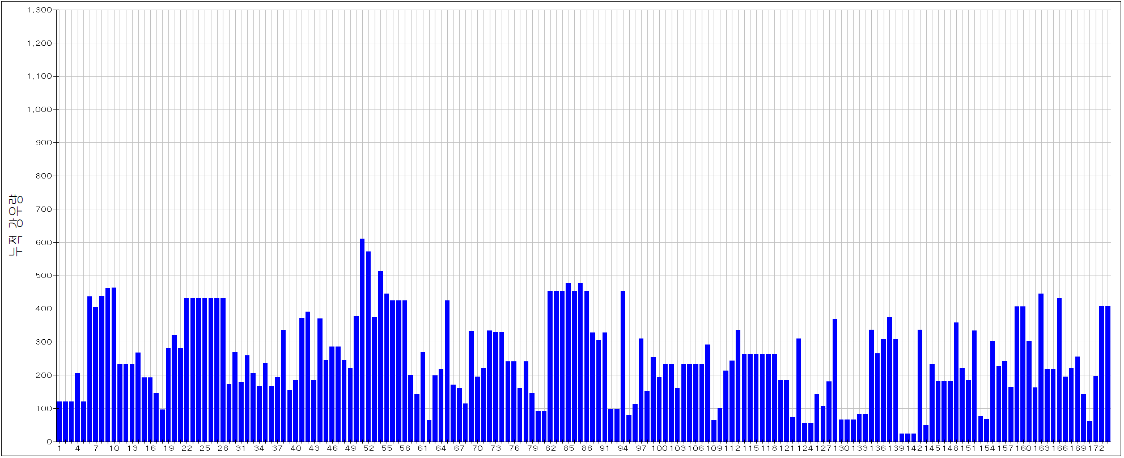

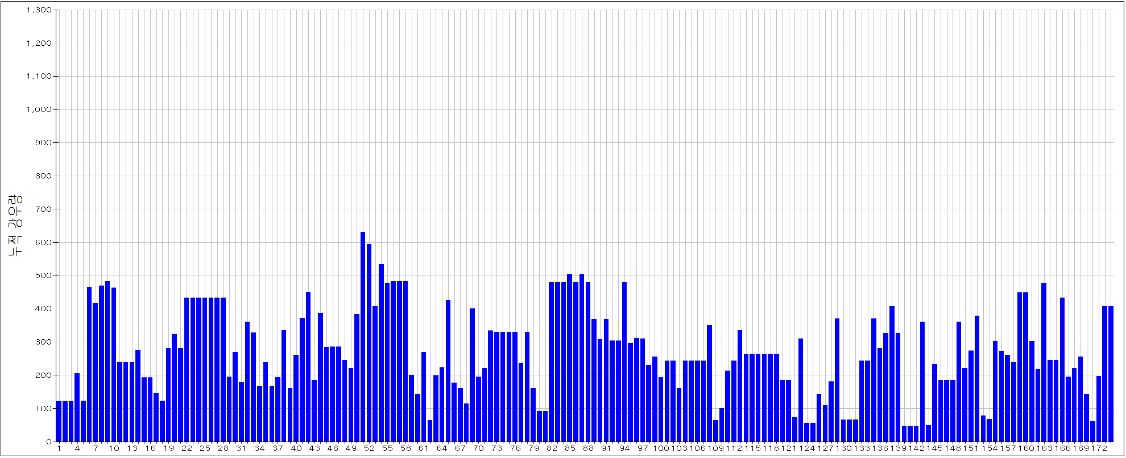

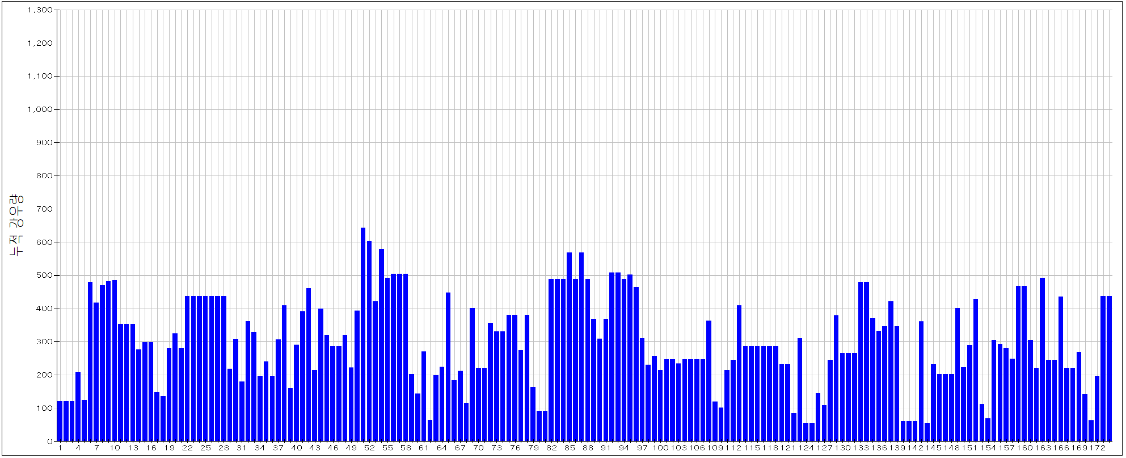


**Eq. (A.5)**


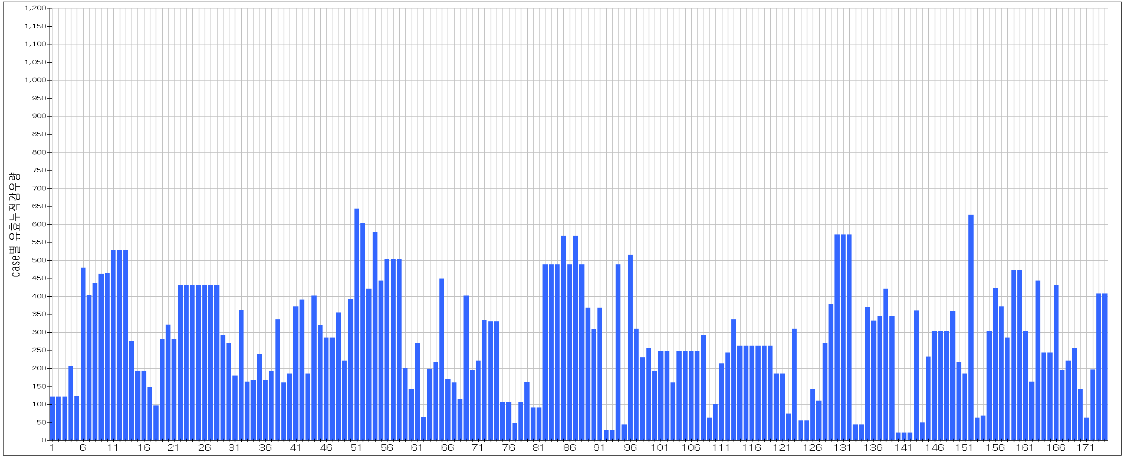

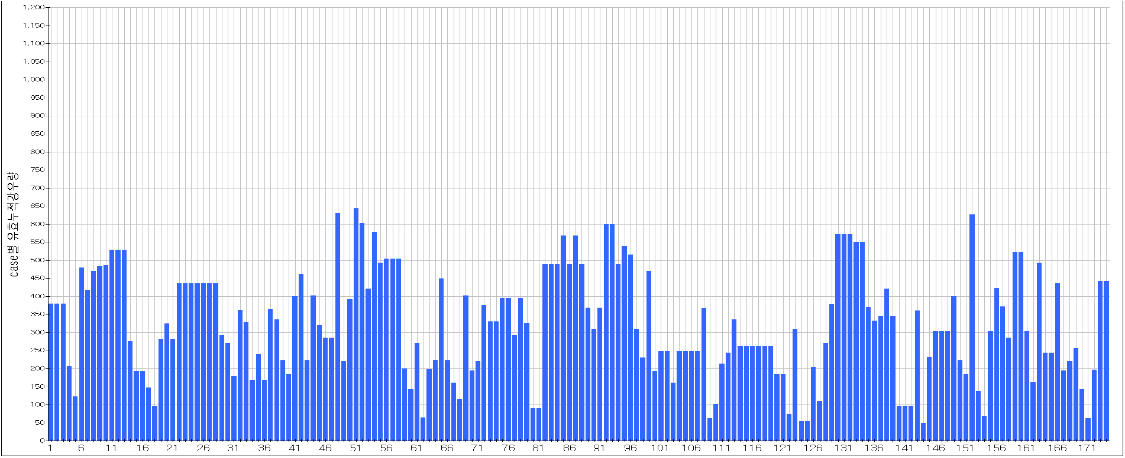

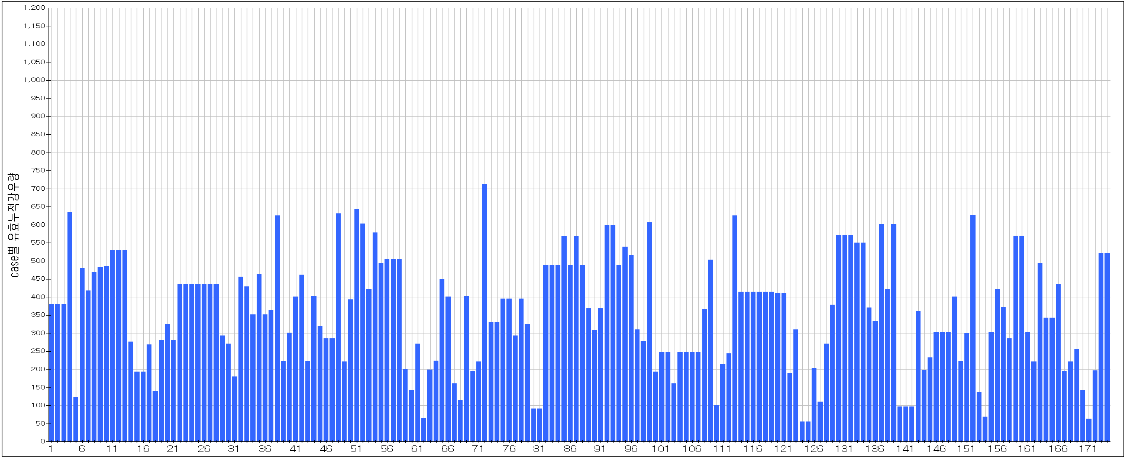

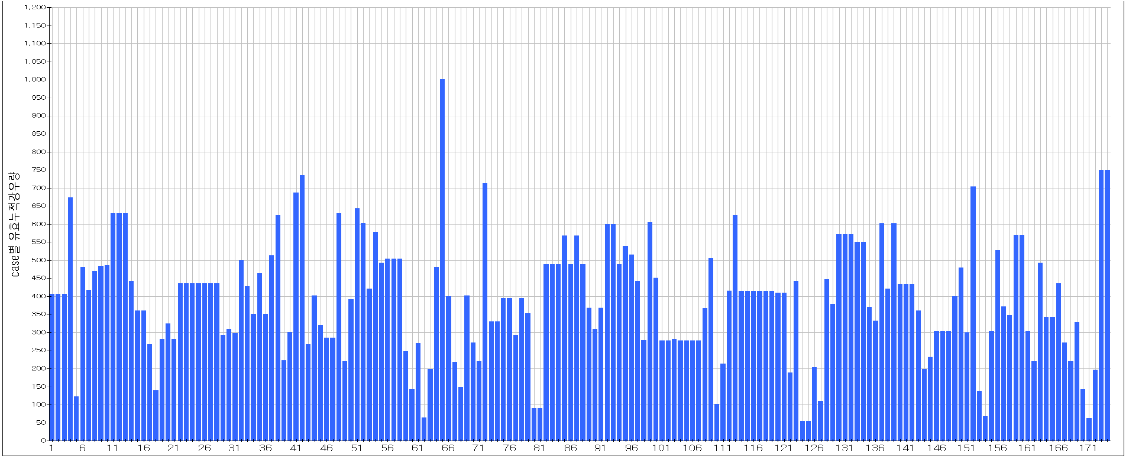

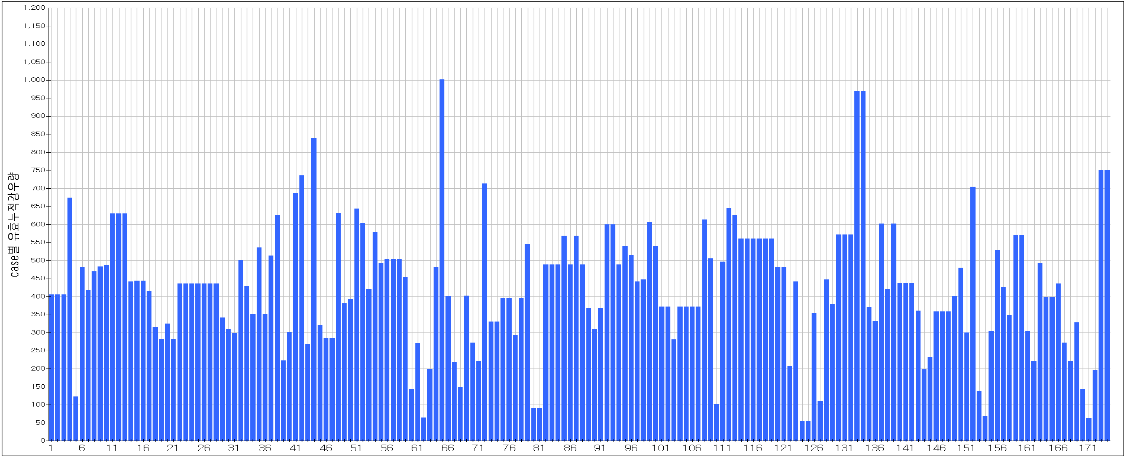

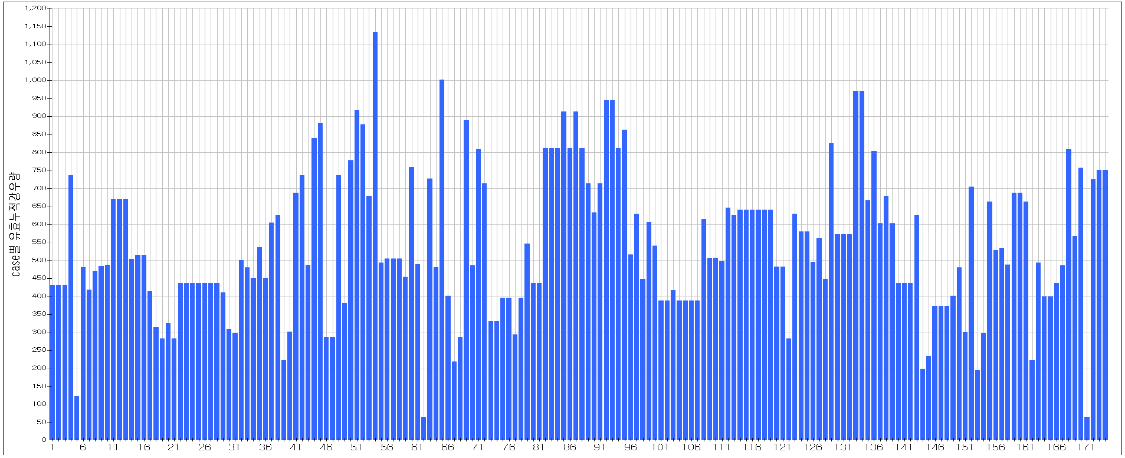


**Eq. (A.6)**

| D_G_ | SP | TWI |
| --- | --- | --- |
| 12 |  |  |
| 24 |  |  |
| 36 |  |  |
| 48 |  |  |
| 672 |  |  |

| D_G_ | TD | PR |
| --- | --- | --- |
| 12 |  |  |
| 24 |  |  |
| 36 |  |  |
| 48 |  |  |
| 672 |  |  |

| D_G_ | FD | SD |
| --- | --- | --- |
| 12 |  |  |
| 24 |  |  |
| 36 |  |  |
| 48 |  |  |
| 672 |  |  |

| D_G_ | FT | ST |
| --- | --- | --- |
| 12 |  |  |
| 24 |  |  |
| 36 |  |  |
| 48 |  |  |
| 672 |  |  |

| D_G_ | DC | AF |
| --- | --- | --- |
| 12 |  |  |
| 24 |  |  |
| 36 |  |  |
| 48 |  |  |
| 672 |  |  |

**Eq. (A.7)**

| IETD | AS | CU |
| --- | --- | --- |
| 12 |  |  |
| 24 |  |  |
| 36 |  |  |
| 48 |  |  |
| 672 |  |  |

| IETD | SP | TWI |
| --- | --- | --- |
| 12 |  |  |
| 24 |  |  |
| 36 |  |  |
| 48 |  |  |
| 672 |  |  |

| IETD | TD | PR |
| --- | --- | --- |
| 12 |  |  |
| 24 |  |  |
| 36 |  |  |
| 48 |  |  |
| 672 |  |  |

| IETD | FD | SD |
| --- | --- | --- |
| 12 |  |  |
| 24 |  |  |
| 36 |  |  |
| 48 |  |  |
| 672 |  |  |

| IETD | FT | ST |
| --- | --- | --- |
| 12 |  |  |
| 24 |  |  |
| 36 |  |  |
| 48 |  |  |
| 672 |  |  |

| IETD | DC | AF |
| --- | --- | --- |
| 12 |  |  |
| 24 |  |  |
| 36 |  |  |
| 48 |  |  |
| 672 |  |  |
